# Divergent functional traits and gene expression profiles in native and encroaching plant species across an alpine elevational gradient

**DOI:** 10.3389/fpls.2025.1656812

**Published:** 2025-10-07

**Authors:** Zhongzan Yang, Jian You, Jiangnan Li, Wei Zhao, Ming Xing, Yujiao Zhang, Ma Cui, Yuqiao Gong, Yueming Zhao, Xia Chen

**Affiliations:** National and Local United Engineering Laboratory for Chinese Herbal Medicine Breeding and Cultivation, School of Life Sciences, Jilin University, Changchun, Jilin, China

**Keywords:** plant adaptive adjustment, alpine tundra, CSR strategy, transcriptome, Changbai Mountain

## Abstract

**Introduction:**

The examination of plant adaptive responses to their native habitats amid global climate change is a critical research focus. Alpine tundra ecosystems, with extreme conditions (e.g., low temperatures and nutrient scarcity), present unique challenges to plant survival. This study aimed to explore how plants adapt to the alpine tundra environment, comparing native species and an encroaching species.

**Methods:**

We analyzed 10 native alpine tundra plant species and one encroaching species (*Deyeuxia angustifolia*) in the Changbai Mountain region. Our approach combined three methods: Morphological characteristic analysis to assess structural adaptations; CSR strategy evaluation (competitive, stress-tolerator, ruderal strategies) to characterize ecological strategies; Comparative transcriptome analysis to reveal molecular mechanisms of adaptation.

**Results:**

Native dwarf shrubs and herbs: activated defense responses, immune responses, and ubiquitous proteins to cope with thermal and oxidative stress. Evolved distinct pathways to adapt to nitrogen deficiency, cold stimuli, and water scarcity. Key proteins (MYC2, ChiB, PI-PLC, Hsp70, POD) drove stress-tolerator (S-related) strategies. Encroaching species (*D. angustifolia*): efficient adaptation to nitrogen deficiency, tolerance to water deficits, and insensitivity to cold stimuli likely fueled its proliferation in alpine tundra. Transcriptomic insights: traditional stressors (nitrogen deficiency, water deficit, cold) exerted lower transcriptional regulatory pressure on plants than other stressors. Gene expression patterns linked to resource acquisition traits may influence *D. angustifolia’s* ecological niche expansion in the tundra.

**Discussion:**

This study emphasizes the convergence of plant adaptive adjustments in alpine tundra ecosystems. By integrating morphological, ecological, and molecular data, our findings provide new foundational insights into plant responses to harsh environments—critical for predicting community dynamics under climate change in alpine systems.

## 1 Introduction

Global climate change is reshaping alpine tundra ecosystems, where plant adaptive adjustment—the ability to adjust phenotypes, strategies, and gene expression in response to environmental shifts—plays a critical role in species survival and community stability (Bjorkman et al., 2018). This phenomenon presents challenges, such as the introduction of encroaching species and the decline or loss of native species (Steinbauer et al., 2018). Alpine tundra is prevalent in various mountain ranges worldwide, situated above timberline, and is distinguished by brief growing periods, frigid temperatures, nutrient-poor soils, and severe climates (Henry et al., 2022; Körner, 2003; Myers-Smith et al., 2015). There is a notable lack of research on the phenomena of adaptive response of plant species in the tundra, especially on the migration of native herbaceous plants from lower altitudes to the alpine tundra.

The alpine tundra environment of Changbai Mountain is currently experiencing significant transformations. This alpine tundra, which was shaped by the recession of Quaternary glaciers, serves as a notable example of China’s alpine tundra and represents the southernmost extent of this ecosystem in eastern Eurasia (Shen et al., 2015; Wenduo et al., 2004). Dwarf shrubs and herbs predominate the vegetation in the alpine tundra, mirroring similar patterns seen in other alpine tundra regions worldwide. Ecological niche reconfiguration of Changbai Mountain’s alpine tundra has been changing due to the impact of global climate change (Wang et al., 2019b). Previous research have indicated that alpine shrub tundra may be supplanted by herbaceous tundra (Jin et al., 2019), with a transition in dominant species from dwarf shrubs to herbaceous plants in Changbai Mountain (Jin et al., 2018; Wang et al., 2019b), accompanied by the emergence of encroaching species, exemplified by *Deyeuxia angustifolia* (*D. angustifolia*) (Zong et al., 2016a).

The adaptive response of vegetation to a dynamic environment is achieved through a range of strategies. Grime’s (1977) concept of ‘CSR plant strategies’, which categorizes species based on their response to competition, stress, and ruderal conditions, has been widely used to explain global plant adaptability, particularly in challenging environments (Pierce et al., 2017; Yu et al., 2022). However, there is a paucity of research exploring the applicability of the CSR theory in the context of alpine tundra ecosystems. ​The S-strategy (stress-tolerance strategy) is typically adopted by plants in variable and resource-poor environments, primarily investing in defense capabilities to maintain individual survival (Yu et al., 2022; Zhou et al., 2021). These plants may be small or gradually increase in size over a long life cycle.​ The C-strategy (Competitive strategy) is centered on acquiring advantages through competitive interactions in resource-rich environments and the R-strategy (Ruderal strategy) is characterized by rapid growth, reproduction, and the colonization of temporary or disturbed habitats. Collectively, these three strategies constitute a flexible adaptive mechanism enabling plants to cope with variable environmental conditions. Meanwhile, in resource-poor environments, ecological strategies tend to converge toward stress tolerance; however, the adaptive adjustment of plant traits does not permit ecological strategies to shift in response to environmental changes (Soudzilovskaia et al., 2013; Zhang et al., 2024b). Altitudinal gradients serve as a prevalent research approach for alpine species and offer exceptional opportunities for investigating plant adaptive adjustment (Sun et al., 2018; Zhang et al., 2016), as they minimize confounding variations stemming from factors like sunlight exposure, geological characteristics, and biogeographic history (Halbritter et al., 2018). This is particularly advantageous in mountainous regions, where environmental alterations can transpire over relatively short distances and where population dispersal and gene flow are considerable (Bridle and Vines, 2007; Sexton et al., 2014).

**Figure 1:**
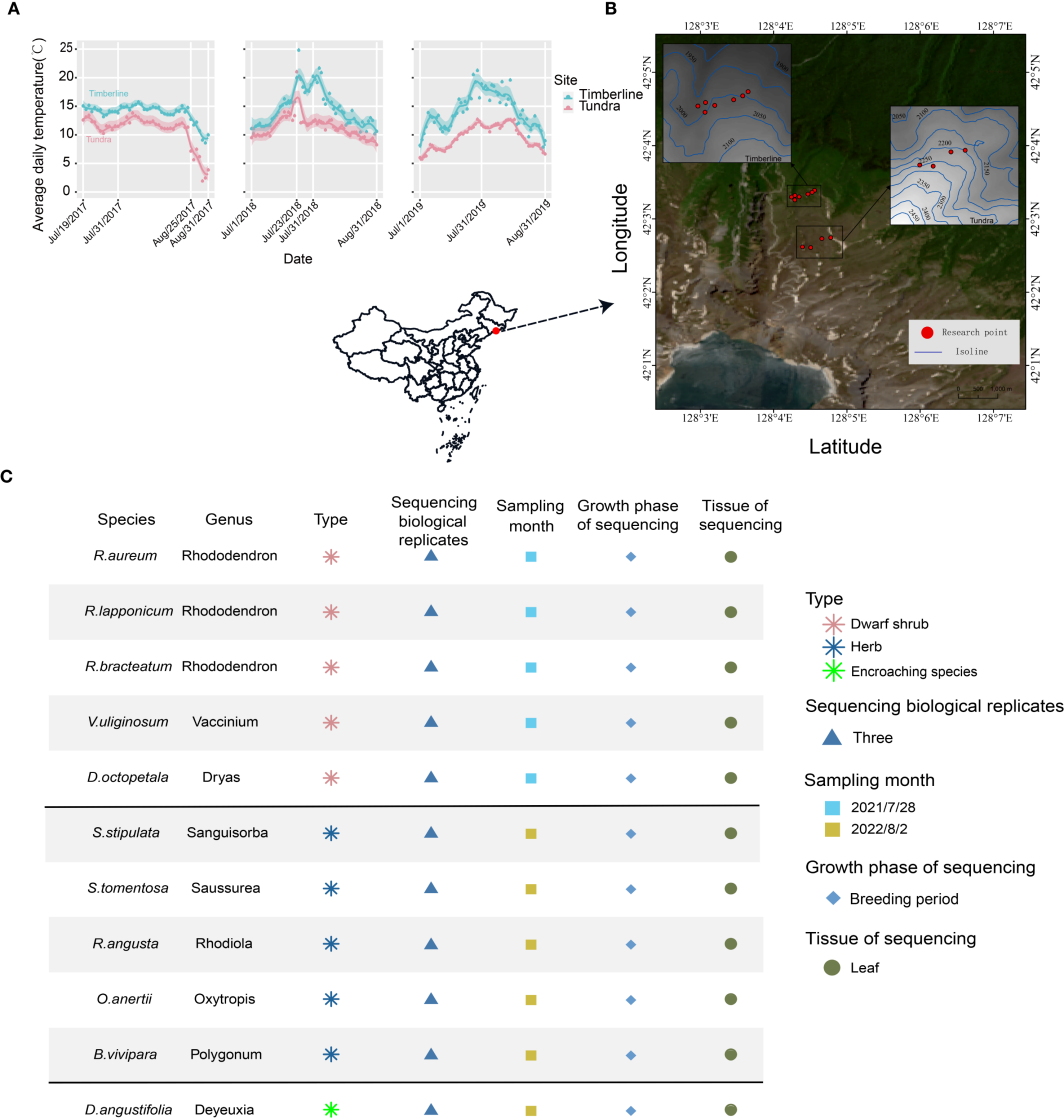
Geographical features of the study area at regional and local scales. **(A)** Average daily soil surface (10cm below ground) temperature of the study area for July and August during 2017-2019. **(B)** Satellite image of the study area showing the location of sampling sites (red dots). **(C)** Overview of the 11 species used in this study. The table describes plant type, sampling site, sampling time, growth phase of sequencing and tissue of sequencing.

Limited research has been conducted in the realm of molecular biology concerning alpine plant species, with a particular emphasis on functional genes unique to individual species (Kubota et al., 2015). Transcriptome analysis uncovers molecular mechanisms, as gene expression patterns bridge genotypes and adaptive phenotypes, revealing how transcriptional regulation supports adaptive responses across environmental gradients (Rivera et al., 2021), playing a key role in responding to environmental fluctuations over both short and long generational time frames (de Nadal et al., 2011; Schlichting and Smith, 2002). Numerous experiments have been conducted to investigate the transcriptomes of sessile organisms (Groen et al., 2020; Nagano et al., 2019; Thomas et al., 2018). To enhance our understanding of plant adaptive response to alpine tundra habitats, a field investigation was carried out in the alpine tundra and timberline regions of Changbai Mountain (as depicted in **Figures 1B, C**). The study focused on examining the CSR strategy and transcriptome control of both native (dwarf shrubs and herbs) and encroaching species (*D. angustifolia*) (Huang and Li, 1984; Li et al., 2023). To minimize habitat variability, plants from the timberline were utilized as an informal control samples. we aim to: (1) identify adaptive response in native species; (2) link morphological traits to adaptive strategies (e.g., S-strategy); and (3) reveal transcriptional regulation underlying these adaptations, and the trait and gene expression shifts of *D. angustifolia* across the gradient differ from the average shift seen in the native species. This design integrates ecological gradient analysis with molecular ecological approaches to bridge macro-scale climate change and micro-scale adaptive phenomena.

## 2 Materials and methods

### 2.1 Study site

Changbai Mountain, located at coordinates 41°41049′′ to 42°25018′′ N and 127°42055′′ to 128°16048′′ E, with an elevation of 2691 meters above sea level, stands as the highest mountain system in northeastern China (**Figure 1B**) (Du et al., 2021; Shen et al., 2014). The region experiences a mean annual temperature of -7.3 °C, rising to 5.9 °C during the average growing season from June to September (Du et al., 2018; Li et al., 2023). With an annual precipitation of approximately 1100 mm, the majority of rainfall is concentrated during this period, reflecting a typical temperate continental climate (Du et al., 2021). The topographic characteristics vary among the four slopes of the mountain, with the northern slope exhibiting a relatively moderate incline (average slope < 3%) and facing away from the prevailing winds (Jin et al., 2018; Shen et al., 2013). Alpine tundra is found at elevations exceeding 2000 m and is particularly well-developed on the upper portion of the volcanic cone at altitudes surpassing 2500 m (Wu et al., 2007). The average daily temperature exhibits variations across vegetation zones, specifically between alpine tundra and timberline (**Figure 1A**, **Supplementary Table 1**). Precipitation and temperature demonstrate a synchronized increase in the months of July and August (**Supplementary Figure 1A**, **Supplementary Table 2**, **Supplementary Figure 1B**). Winds persist in the alpine tundra and timberline regions (**Supplementary Figure 1C**), potentially causing significant wind damage (Jin et al., 2021), and low soil moisture content (**Supplementary Figure 1D**).

The preservation of the natural vegetation of Changbai Mountain is effectively maintained due to the establishment of the Changbai Mountain National Nature Reserve in the 1960s. This reserve strictly prohibits human activities such as logging, hunting, and plant gathering, thereby preventing anthropogenic causes of herb encroachment into the alpine tundra ecosystem (McDougall et al., 2011). Common vegetation in the area includes low stature or prostrate shrubs such as *Rhododendron aureum* Georgi (*R. aureum*), *Rhododendron lapponicum* (L.) Wahl. (*R. lapponicum*), *Rhododendron bracteatum* Rehd. et Wils. (*R. bracteatum*), *Vaccinium uliginosum* L. (*V. uliginosum*), *Dryas octopetala* L. (*D. octopetala*), as well as herbaceous species like *Sanguisorba stipulata* Rafinesque (*S. stipulata*), *Bistorta vivipara* (L.) Gray. (*B. vivipara*), *Saussurea tomentosa* Kom. (*S. tomentosa*), *Rhodiola angusta* Nakai (*R. angusta*), *Oxytropis anertii* Nakai ex Kitag. (*O. anertii*), and *D. angustifolia* (Huang and Li, 1984; Jin et al., 2018; Wang et al., 2019b; Zong et al., 2016b).

### 2.2 Experimental design and sampling

#### 2.2.1 Sampling sites and experimental grouping

The sampling areas in this study included two locations: alpine tundra: 42.043°-42.045° N, 128.073°-128.079° E, at an elevation of 2250 ± 25 m; timberline: 42.054°-42.056° N, 128.070°-128.075° E, at an elevation of 2000 ± 20 m (**Figure 1B**). The experimental design adopted a “cross-habitat comparison within the same species” approach: populations in the timberline served as an informal control group, while populations in the alpine tundra were designated as the experimental group. Representative sampling sites were identified through horizontal sampling (**Figure 1C**, **Supplementary Figure 2**).

#### 2.2.2 Sampling selection, sampling time, and replication

A total of 11 species were selected based on vegetation dominance, encompassing dwarf shrubs, herbaceous plants, and encroaching species (*D. angustifolia*). All samples were collected synchronously in both habitats from 09:00 to 11:00 on the same day (to minimize transcriptional fluctuations induced by intense midday sunlight). Climatic conditions at the sampling sites on the collection day are provided in **Supplementary Table 3**. For this study, sample collection was structured to ensure robustness across different analytical needs. For baseline samples, at least 15 biological replicates (individual plants) of each species were gathered from both the alpine tundra and timberline habitats, providing a foundational dataset for broader ecological comparisons. When it came to morphological measurements, mature and non-senescent leaves were sampled from a minimum of 6 individuals per species; this approach balanced practicality with the need to capture intraspecific morphological variability. For sequencing-related analyses, 3 biological replicates were selected for each species in both habitats. In total, this resulted in 66 samples. Specifically, there were 60 libraries prepared for native species and 6 libraries for the encroaching species *D. angustifolia*, with each of these libraries further including 3 biological replicates to account for potential variability in sequencing workflows.

#### 2.2.3 Sampling methods and preservation

Healthy, fully developed, and non-senescent leaves produced in the current year were collected and processed into two groups: sequencing samples were immediately frozen in liquid nitrogen and stored at -80 °C; morphological measurement samples were preserved with ice packs and subjected to leaf morphological measurements immediately after descending the mountain. No sample pooling was performed, and all analyses were conducted independently based on individual biological replicates.

### 2.3 *De novo*-seq analysis and data processing

#### 2.3.1 RNA extraction and quality control

Total RNA was extracted from leaves using a TRIzol kit (Invitrogen, Carlsbad, CA, USA) and treated with 10 units of DNase I (Takara, Dalian, China) at 37 °C for 30 min to eliminate genomic DNA. The quality of the RNA was assessed using an Agilent 2100 Bioanalyzer with the Agilent RNA 6000 Nano Kit.

#### 2.3.2 cDNA library construction and sequencing

A total of 20 μg of RNA with a RNA Integrity Number (RIN) greater than 8.0 was used to construct a cDNA library, with Oligo (dt) selection employed to enrich poly (A) mRNAs for each sample. The mRNA underwent fragmentation and reverse transcription using N6 random primers to generate double-stranded cDNA. The resulting cDNA was then subjected to end-repair and 3′ adenylation, followed by ligation of adaptors to the adenylated cDNA fragments. The ligation products were purified and subjected to multiple rounds of PCR amplification to enrich the cDNA template. Subsequently, the PCR products were denatured by heat and the single-stranded DNA was looped through splicing oligonucleotides and DNA ligase. A total of 60 libraries, with 3 replicates per sample, were sequenced for native species, while 6 libraries, each with 3 replicates of *D. angustifolia*, were sequenced for encroaching species. Sequencing was conducted in pairs (2 × 100 bp) on the BGISEQ-500 sequencing platform for native species and on the Illumina NovaSeq 6000 for encroaching species.

#### 2.3.3 Raw data processing and transcriptome assembly

The raw data underwent filtering using SOAPnuke (v1.4.0) (Chen et al., 2017), followed by assembly of clean reads using Trinity (v2.0.6) and assessment of assembly quality using BUSCO (Haas et al., 2013). The assembled unique gene was aligned with clean data using Bowtie 2 (v2.2.5) and gene expression levels were determined using RSEM (v1.2.8) (Langmead and Salzberg, 2012; Li and Dewey, 2011).

#### 2.3.4 Functional annotation and differential expression analysis

Gene functions were annotated using various databases including NCBI non-redundant protein sequences (Nr), protein family (Pfam), clusters of Orthologous Groups of proteins (KOG/COG/EggNOG), Swiss-Prot, Kyoto Encyclopedia of Genes and Genomes (KEGG), and Gene Ontology (GO). All differentially expressed genes (DEGs) in alpine tundra samples were identified based on direct comparisons within the same species against an informal control group (samples from timberline). Differential gene analysis within groups was conducted utilizing DESeq2 with the criteria of |log2 (Fold Change)| ≥ 2 and adjusted P value ≤ 0.05 (Love et al., 2014). Subsequently, DEGs were functionally categorized based on GO and KEGG annotation results and official classifications. KEGG enrichment analysis was performed using the phyper function in R software (Kanehisa et al., 2017), while GO enrichment analysis was conducted using the TermFinder package (Boyle et al., 2004). Candidate genes meeting the threshold of a Q value ≤ 0.05 were deemed significantly enriched. Gene families were assembled using OrthoFinder (Emms and Kelly, 2019), and the phylogenetic tree was constructed using the OrthoFinder rooted species tree with the ‘ggtree’ and ‘treeio’ packages in R (Wang et al., 2020; Yu et al., 2017).

#### 2.3.5 RT-qPCR validation

cDNA synthesis was performed following the protocol of the MonScript™ RTIII All-in-One Mix with dsDNase kit (Monad Biotech Co. Ltd., Wuhan, China), and RT-qPCR was conducted according to the instructions of the MonAmp™ ChemoHS qPCR Mix (Monad Biotech Co. Ltd., Wuhan, China). The fluorescence quantitative results were calculated using Excel software to determine the normalized gene expression’s 2^-ΔΔCt^ value in relation to the expression of the gene *β*-actin. The primers utilized for reverse transcription quantitative polymerase chain reaction (RT-qPCR) were documented in **Supplementary Table 4**.

### 2.4 Data collection and analyses

Plant height was assessed on the hill directly. Mature but non-senescent leaves were sampled from a minimum of six individual plants per species, adhering to the standardized protocols outlined by Harguindeguy et al (Pérez-Harguindeguy et al., 2013). Leaf area (LA) was quantified using a Li-3000 leaf area meter (Li-Cor, Inc, Lincoln, NE, USA), while leaf dry weight (LDW) and leaf fresh weight (LFW) were also measured. These measurements were utilized to calculate CSR strategy (CSR scores) using the spreadsheet calculation tool ‘StrateFy’ (Pierce et al., 2017), which has been widely adopted (Dayrell et al., 2018; Escobedo et al., 2021; Yu et al., 2022). Fresh leaves were weighed using an analytical balance (ME104E, Mettler Toledo) to determine leaf fresh weight (LFW). Subsequently, the leaves were subjected to oven-drying at 65 °C for 48 hours until a constant weight was achieved, and the dry weight (LDW) was recorded with a precision of 0.001 g. Additionally, five leaf traits, including leaf succulence index (LSI), leaf water content (LWC), leaf mass per area (LMA), leaf dry matter content (LDMC), and specific leaf area (SLA), as well as the CSR strategy, were calculated using the ‘StrateFy’ method, which has been extensively validated through experimental research (Dayrell et al., 2018; Pierce et al., 2017; Wang et al., 2018). Soil temperature was monitored using a soil temperature and humidity recorder (L99-TWS-1, Fotel Precise Instrument Co., Ltd., Shanghai, China) at alpine tundra (42.043471° N, 128.075093° E) and timberline (42.055019° N, 128.072465° E), and the soil moisture dataset is sourced from the National Tibetan Plateau/Third Pole Environment Data Center, with the CSTR (China Science and Technology Resource Identifier) accession number: https://cstr.cn/18406.11.Hydro.tpdc.271762. Statistical analyses were conducted using R software (version 4.3.1, http://www.r-project.org). Climate data were obtained from WorldClim (www.worldclim.org) and processed using R (package: raster) (Hijmans and Van Etten, 2022). Principal component analysis (PCA) was performed using R (package: FactoMineR) (Le et al., 2008), while random forest (RF) analysis was conducted using R (package: rfPermute) (Archer, 2021). K-medoids clustering analysis was performed using R (package: cluster and fpc) (Hennig, 2022; Maechler et al., 2022). The Volcano plot was generated using the R programming language with the ggrepel and ggplot2 packages (Ginestet, 2011). The circular Volcano plot was created using the ‘scRNAtoolVis’ package developed by Junjun Lab, available at https://github.com/junjunlab/scRNAtoolVIS. The UpSet plot was produced using the UpSetR package in R (Lex et al., 2014).

## 3 Results

### 3.1 Phenotypic analysis of native species in alpine tundra

**Figure 2:**
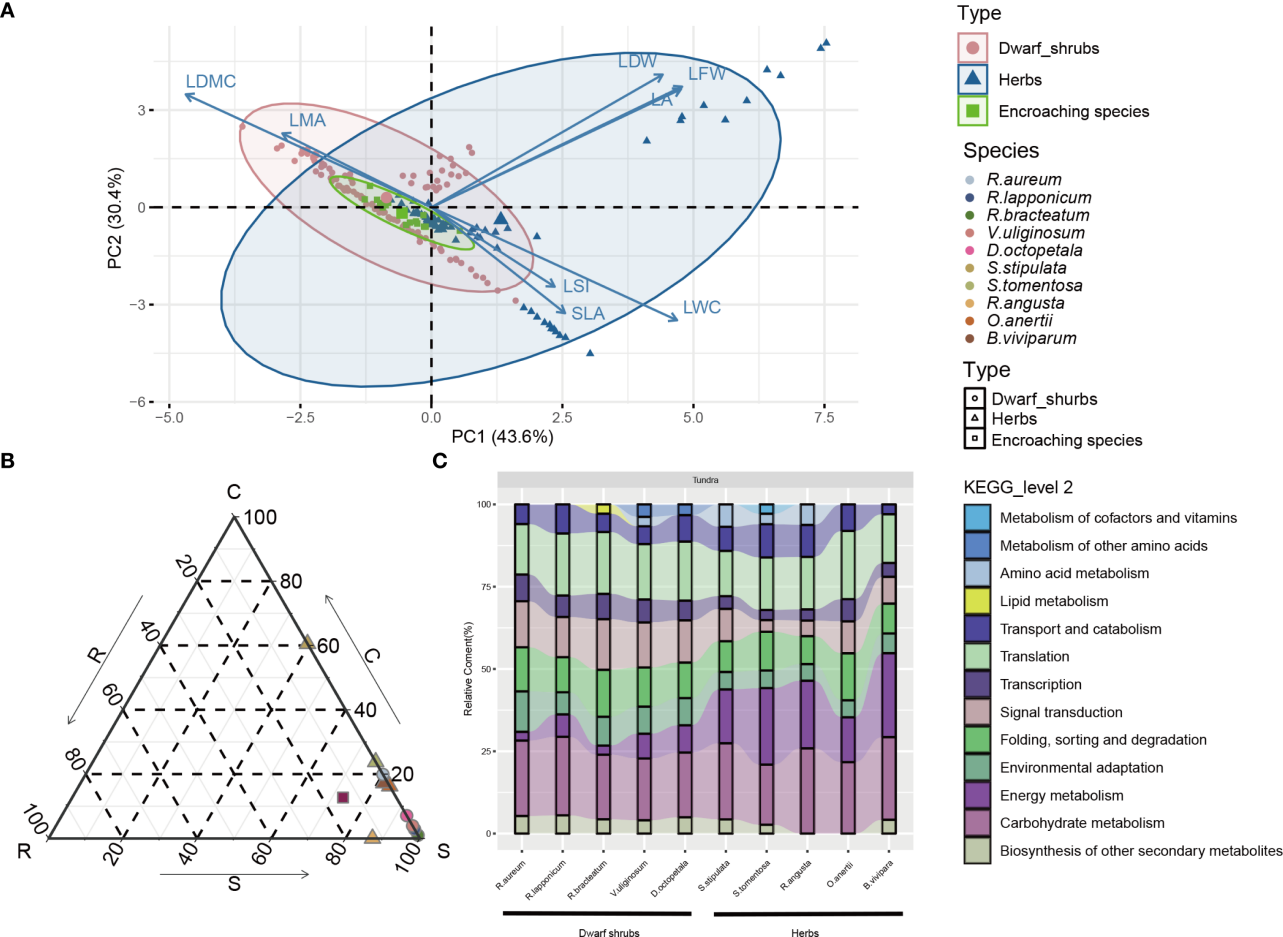
**(A)** Principal component analysis (PCA) of leaf traits of different plant types in alpine tundra. Pearson’s correlation coefficients between the PCA axes (PC1 and PC2) and leaf traits are shown positively or negatively. LA, LFW, LDW, LSI, LMA, LDMC, SLA and LWC denote leaf area, leaf fresh weight, leaf dry weight, leaf succulence index, leaf mass per area, leaf dry matter content, specific leaf area and leaf water content, respectively. Points represent individuals and inertia ellipses are placed in 95% **(B)** Ternary plot of functional CSR-type differences of plants in alpine tundra. **(C)** KEGG Level 2 pathways annotations of top 15 tertiary KEGG pathways (removing large pathways such as ‘Metabolic pathways (ko01100)’, ‘Biosynthesis of secondary metabolites (ko01110)’, ‘Carbon metabolism (ko01200)’, ‘Biosynthesis of amino acids (ko01230)’, ‘Biosynthesis of cofactors (ko01240)’) for genes in groups of highest FPKM values. Genes in group of highest FPKM values are generated on first clustered into 5 clusters by K-medoids and then Pearson correlation hierarchical clustering grouped into 3 groups in which involving in highest FPKM values’ group.

The phylogenetic analysis demonstrated that the ten species of common tundra native plants were categorized into two groups: dwarf shrub and herb, originating from eight genera (**Supplementary Figure 3**). Remarkably, there were notable differences in plant height and LA between the tundra and timberline environments, as indicated by the t-test (*p*<0.05) (**Supplementary Figures 1E, F**; **Supplementary Tables 5**, **6**). Specifically, the native plants in alpine tundra displayed reduced stature and smaller LA in comparison to those in timberline habitats. Principal component analysis (PCA) revealed that there was no significant variation in leaf traits among dwarf shrubs, herbs, and encroaching species, with smaller projections of SLA in principal component 1 (PC1) and principal component 2 (PC2) (**Figure 2A**). Additionally, the presence of plant strategies based on the CSR strategy was observed (**Figure 2B**, **Supplementary Table 7**). In the alpine tundra ecosystem, dwarf shrubs, herbs, and neo-colonizer predominantly adopted strategies falling within the Stress-tolerator (S) or S-related categories.

### 3.2 Molecular biological analysis of native plants in alpine tundra

#### 3.2.1 Analysis of genes expression in native species

The group exhibiting the highest FPKM value (**Supplementary Figures 4B, C**) resulted from k-medoids clustering of genes from alpine tundra species obtained through *de novo* sequencing (**Supplementary Table 8**) and was subjected to GO annotation (**Supplementary Figures 4D, E**; **Supplementary Tables 9**, **10**). Among various habitat groups of vegetation types, second-level annotations associated with ‘response to stimulus’ ranked within the top six in terms of annotation volume within the level 1 GO term ‘biological process’ (**Supplementary Figures 4D, E**; **Supplementary Tables 9**, **10**). Regarding KEGG annotations, the percentage of KEGG level 2 results (**Figure 2C**, **Supplementary Table 11**) does not provide sufficient explanation for the utilization of biased S-associated strategies in native plants (dwarf shrubs and herbs). Therefore, additional analyses of DEGs were performed.

#### 3.2.2 Analysis of native species’ DEGs

**Figure 3:**
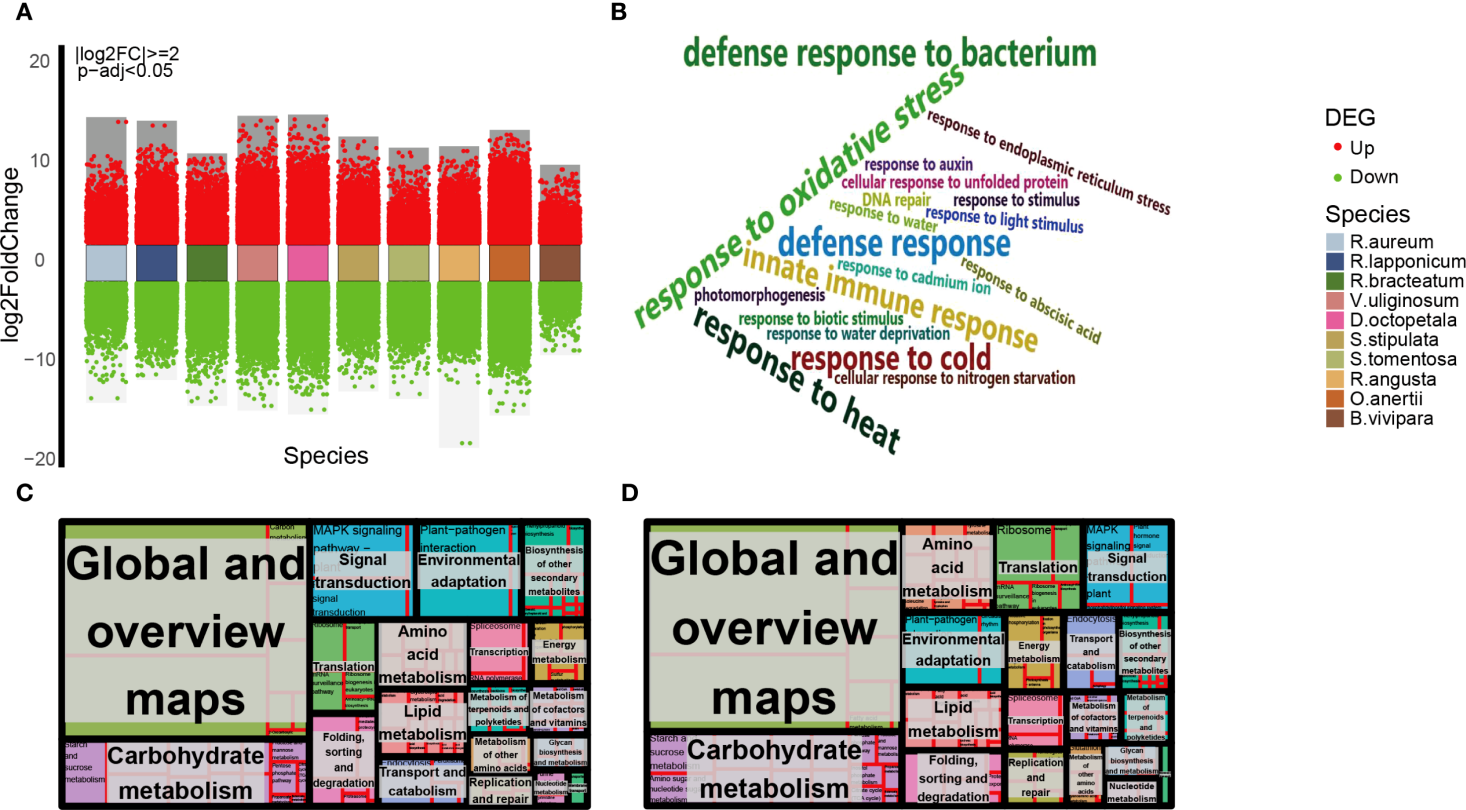
Overview of DEGs of native plants in alpine tundra assemblies and annotations. **(A)** Differential gene expression analysis showing up- and down-regulated genes across 10 native plants. Volcano plots showed FDR and log2FC for DEGs which are significantly upregulated (red dots) or downregulated (green dots). **(B)** Word cloud shows intersections of 10 native species’ DEGs with level 3 annotations in GO level 2 annotations. Name sizes are randomized. Results of no significant difference in the percentage of DEGs annotations within the KEGG enrichment analysis groups of dwarf shrub **(C)** and herb **(D)** generated in R using the package ‘treemap’ (https://cran.r-project.org/web/packages/treemap/index.html). the analysis of KEGG level 3 pathways annotations did not reveal any significant differences between species within the groups of dwarf shrubs and herbs (Pearson’s chi-squared test, *p* > 0.05). Each rectangle is a single cluster representative. The representatives are joined into ‘superclusters’ of loosely related pathways, visualized with different color. The size of the rectangles is adjusted to reflect the percentage.

A significant number of DEGs, ranging from 3990 to 24385, were annotated for each species (**Figure 3A**, **Supplementary Table 12**). A substantial proportion of these DEGs are categorized under the secondary pathway ‘response to stimulus’ within the primary annotation ‘biological process’, as indicated in **Supplementary Figures 5A and B** (**Supplementary Tables 13**, **14**). This observation is consistent with the annotation results derived from the Pearson correlation hierarchical clustering analysis utilizing K-medoids (**Supplementary Figures 4D, E**). To delve deeper into the unique plant adaptive adjustment in tundra environments, an analysis was performed on the intersection of GO annotation at level 3 within the second level GO term ‘response to stimulus’ for 10 indigenous species (**Supplementary Figure 5C**). The analysis identified 19 frequently annotated Gene Ontology terms by intersection, as depicted in **Figure 3B** and **Supplementary Table 15**. These terms encompassed categories such as ‘defense response (GO:0006952)’, ‘innate immune response (GO:0045087)’, ‘response to oxidative stress (GO:0006979)’, ‘response to water deprivation (GO:0009414)’, ‘response to cold (GO:0009409)’, ‘response to heat (GO:0009408)’, and ‘defense response to bacterium (GO:0042742)’. To advance the examination of plant adaptive response to alpine tundra environments, pertinent pathways associated with environmental adaptation and signaling signal transduction were viewed, as illustrated in **Figures 3C** and D (**Supplementary Table 16**). These pathways encompass ‘plant-pathogen interaction (ko04626)’, ‘MAPK signaling pathway-plant (ko04016)’, ‘plant hormone signal transduction (ko04075)’, and ‘protein processing in endoplasmic reticulum (ko04141)’, which play a role in plants’ responsiveness to stimuli and their adaptive modifications in alpine tundra settings. The relationship of containment and inclusion is evident in the overlap of GO level 3 annotations among ten species. Specifically, an analysis of the intersection of KEGG annotations in these species for DEGs annotated with the GO term ‘defense response (GO:0006952)’ reveals that all are also annotated to ‘plant pathogen interaction (ko04626)’, ‘MAPK signaling pathway-plant (ko04016)’, ‘plant hormone signal transduction (ko04075)’, and various metabolic pathways (**Supplementary Figure 6A**, **Supplementary Table 17**).

#### 3.2.3 Partial defense responses of native plants in alpine tundra

**Figure 4:**
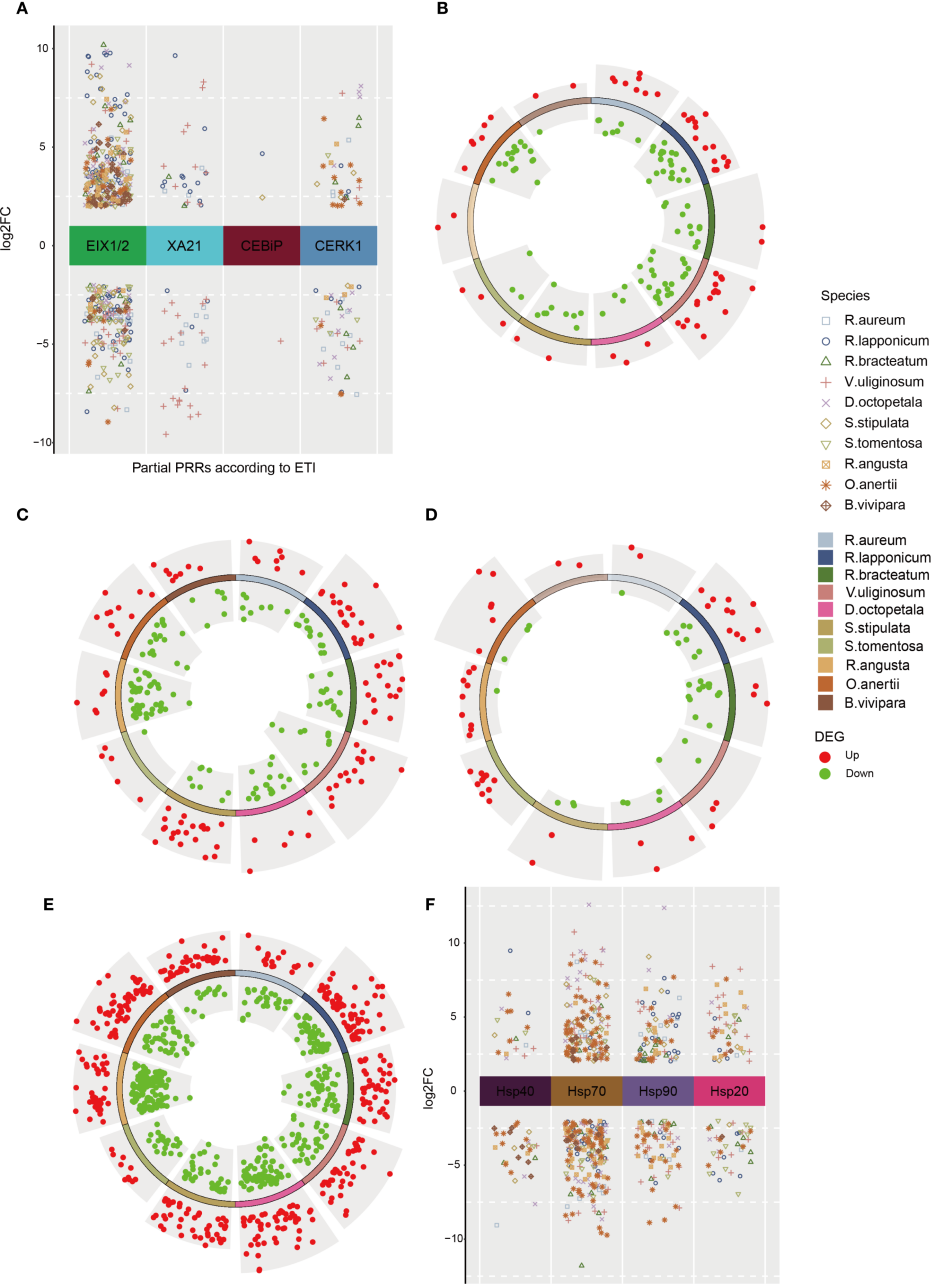
DEGs for proteins related to defense responses across 10 native plants in tundra. **(A)** Partial PRRs’ DEGs according to plant-microbial interaction in native species. Circle volcano plot shows DEGs which are significantly upregulated (red dots) or downregulated (green dots) in 10 native species for MYC2 **(B)**, CHiB **(C)**, PI-PLC **(D)**, POD **(E)**. **(F)** DEGs for heat shock proteins (HSPs) in 10 native species.

In the ‘plant pathogen interaction (ko04626)’ pathway, the FLS2 receptors and EFR receptors, which identify the bacterial peptide epitopes flg22 (derived from flagellin) and elf18 (derived from Elongation Factor Tu) respectively, in conjunction with their co-receptor BAK1, demonstrated significant modulation of DEGs in both up- and down-regulated states (**Supplementary Figure 6B**). Similarly, various pattern recognition receptors (PRRs) exhibited diverse numbers of DEGs that were either up- or down-regulated (**Figure 4A**, **Supplementary Table 18**), including EIX1/2. To confirm these results across different species, RT-qPCR analysis was conducted (**Supplementary Figure 8C**). The down-regulated DEGs annotated as PRRs may be associated with the reallocation of limited resources from immunity to essential survival processes in plants (Snoeck et al., 2025). Notably, during field sampling, no visible phenotypic evidence of pathogen infection (e.g., disease spots, wilting) or disease symptoms was observed for any species across all alpine tundra sampling sites. The activation of immune-related pathways thus likely reflects generalized stress adaptation in this harsh environment, aligning with transcriptomic signatures of stress response coordination (Zhu, 2016).

The upregulation of some defensive DEGs (**Supplementary Figure 7A**, **Supplementary Table 19**) associated with pathogen-associated molecular patterns (PAMPs)-triggered immunity (PTI) was observed, with FRK1 identified as having increased gene expression in both dwarf shrubs and herbs. To evade PTI, some microbes deliver effectors to the plant apoplast or directly into cells (Tabassum and Blilou, 2022), thereby inducing effector-triggered immunity (ETI) (**Supplementary Figure 8A**), or even hypersensitive response (HR) (**Supplementary Figure 7B**). Furthermore, the upregulation of relevant DEGs resulted in the expression of KCS (3-ketoacyl-CoA synthase) (**Supplementary Figure 8B**, **Supplementary Table 20**), which is involved in fatty acid synthesis and elongation (Chen et al., 2011b), potentially serving to inhibit HR. The activation of the transcription factor MYC2, which is regulated by jasmonic acid (JA), was observed to modulate a significant number of DEGs in basic endochitinase B (ChiB) (**Figure 4C**, **Supplementary Table 22**), as depicted in **Figure 4B** and **Supplementary Table 21**. MYC2 exerts its influence by antagonistically controlling two distinct branches of the JA signaling pathway, which are responsible for regulating resistance against pests and pathogens (Kazan and Manners, 2013; Wang et al., 2019a). ChiB, along with its subsequent impacts, plays crucial roles in a range of biotic and abiotic defense response, such as antimicrobial, anthelmintic, and responses to cold and heat stimuli (Grover, 2012; Zhou et al., 2020). The downregulation of MYC2 and ChiB DEGs may orchestrate resource reallocation by suppressing energy-intensive immunity, thereby preventing over-defense and synergistically enhancing cold adaptation (Liu et al., 2025; Song et al., 2014). In contrast, some of the genes encoding phosphatidylinositol-specific PLC (PI-PLC) were consistently found to be upregulated in the ‘phosphatidylinositol signaling system (ko04070)’ as depicted in **Figure 4D** and **Supplementary Table 23**. Moreover, PI-PLC demonstrates potential cellular and physiological roles in response to various abiotic and biotic stimuli, including involvement in microbial immunity, addressing nutrient deficiencies, and influencing plant growth and development (Hong et al., 2016; Singh et al., 2015). Subsequently, RT-qPCR validation was performed on the upregulated genes MYC2, ChiB, and PI-PLC across ten different species, as shown in **Supplementary Figures 9A, B, and C**.

#### 3.2.4 Native plants respond to oxidative stress

A significant number of DEGs were found to be annotated to GO term ‘response to oxidative stress (GO:0006979)’. To further investigate these DEGs, KEGG annotations were performed on 10 species, resulting in the identification of intersections with secondary biosynthetic and metabolic pathways (**Supplementary Figure 9D**, **Supplementary Table 24**). Specifically, these DEGs were primarily annotated to four pathways: ‘tryptophan metabolism (ko00380)’, ‘phenylpropanoid biosynthesis (ko00940)’, ‘glutathione metabolism (ko00480)’, and ‘arachidonic acid metabolism (ko00590)’. These pathways were associated with the enzymes CAT (catalase) (**Supplementary Figure 10B**, **Supplementary Table 25**), POD (peroxidase) (**Figure 4E**, **Supplementary Table 26**), and GPX (glutathione peroxidase) (**Supplementary Figure 10C**, **Supplementary Table 27**), respectively. The concurrent upregulation and downregulation of POD gene expression reflects multi-tiered adaptation strategies in plants under extreme environmental stress. The up-regulation of DEGs was observed only in POD genes across all 10 native species, as confirmed by RT-qPCR analysis (**Supplementary Figure 10A**).

#### 3.2.5 Native plants respond to thermal stimuli and nitrogen starvation

The expression of heat shock proteins (HSPs) was induced by heat stimuli, specifically ER-associated degradation (ERAD) (Walter and Ron, 2011; Yoo et al., 2022). The DEGs for Hsp40, Hsp70, Hsp90, and Hsp20 exhibited varying degrees of expression across different species, as shown in **Figure 4F** (**Supplementary Table 28**). Notably, Hsp70 was consistently up-regulated in all species and was subjected to RT-qPCR validation, as depicted in **Supplementary Figure 10D**. HSFs, known as heat stress transcriptional factors, serve as molecular chaperones *in vivo* to prevent protein denaturation and maintain protein homeostasis during stress (Yoo et al., 2022). These HSFs are released from their inhibited state with Hsp70 and Hsp90 under heat stress, allowing them to bind misfolded proteins caused by the same stress (Scharf et al., 2012). Notably, plants diverge from animals in that animal-derived DMAPs may induce HSPs activation (Choi and Klessig, 2016), whereas there is currently no substantiated proof that plant-originated DMAPs can elicit HSPs activation. Moreover, it is suggested that heat shock proteins are primarily associated with heat stimuli rather than other forms of stimuli (Chaudhry and Sidhu, 2022).

A limited number of DEGs were identified across all species categorized under the Gene Ontology tertiary annotation ‘cellular response to nitrogen starvation’ (**Supplementary Figure 10E**, **Supplementary Table 29**). The majority of these DEGs were linked to autophagy, with the exception of *R. aureum*, where they were associated with GABA(A) receptor-associated protein (ATG8), a protein class connected to nitrogen deficiency (Zhang et al., 2022). Interestingly, the up-regulated genes in *R. aureum* for nitrogen starvation were not related to autophagy but rather to allantoinase (allB), an enzyme responsible for converting allantoin, a nitrogenous waste product, into urea, a nitrogen source that can be utilized by plants (Yang and Han, 2004).

#### 3.2.6 Native plants respond to cold stimuli and water deprivation

The vegetation may be subjected to cold stimuli and water deprivation, leading to categorization using GO terms. **Figure 3B** demonstrates that 10 species’ DEGs were consistently annotated to these GO terms. It is important to note that while some DEGs were annotated to the GO terms ‘response to cold (GO:0009409)’ and ‘response to water deprivation (GO:0009414)’, not all exhibited up-regulation, with only a minimal amount showing down-regulation (**Supplementary Figures 11A, B**; **Supplementary Tables 30**, **31**), as seen in *R. lapponicum* and *S. tomentosa*. DEGs in *R. lapponicum* and *S. tomentosa* did not exhibit specific down-regulation associated with cold stimuli compared to plants in timberline (**Supplementary Table 32**), indicating that not all plant species necessarily respond to cold stimuli. The down-regulated DEGs display diversity (Saijo and Loo, 2020), such as the negative regulation of natural immunity by DDX19 (Zhang et al., 2019).

In light of water scarcity, it was observed that among native species, only *S.tomentosa* did not exhibit up-regulation of DEGs (**Supplementary Table 33**). Furthermore, the down-regulated DEGs were annotated as zeaxanthin epoxidase (ZEP), which showed decreased expression in response to drought (Schwarz et al., 2015), suggesting distinct coping ways among native species in the face of water scarcity. Additionally, the lack of a common intersection in the KEGG pathway annotations of DEGs identified through individual GO terms (**Supplementary Figure 11B**) indicates varying degrees of tolerance to water deficit in plants. The same also suggests that the necessity of plant responses to cold stimuli is not necessarily consistent (**Supplementary Figure 11A**).

### 3.3 Adaptation of encroaching species (*D. angustifolia*) to alpine tundra environment

**Figure 5:**
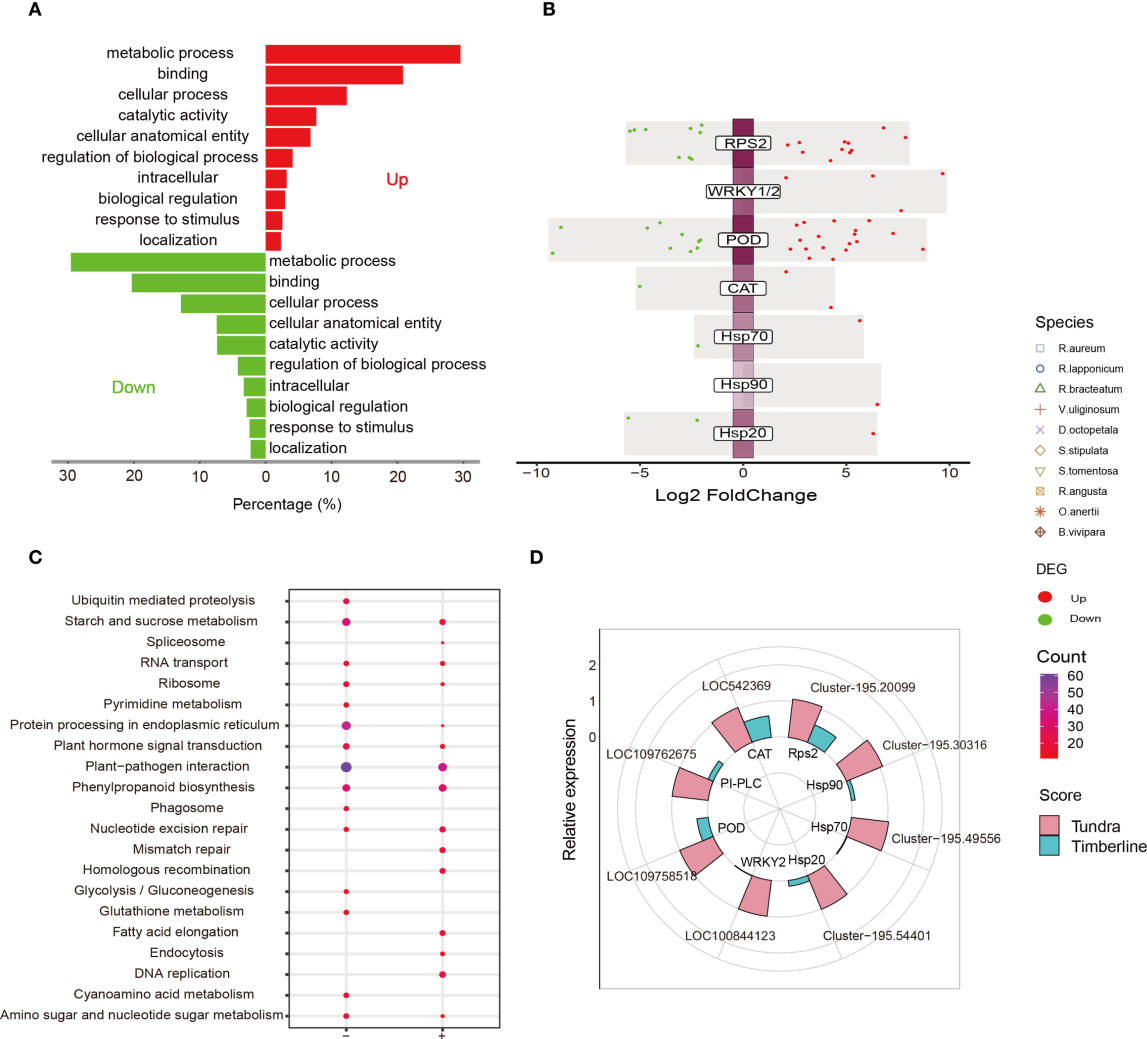
Multidimensional analysis of DEGs in *D*. *angustifolia*. **(A)** GO term analysis of downregulated and upregulated (reference threshold: |log2 FC| ≥ 2; adjusted *p* values < 0.05) genes were performed. **(B)** Differential gene expression analysis showing up- and down-regulated genes across and proteins for self-regulated defense responses. **(C)** KEGG pathways analysis of *D*. *angustifolia*’s up- (+) and down-regulated (-) DEGs, respectively. **(D)** Relative expression of the genes related to some of the resistance proteins by RT-qPCR.

*De novo* sequencing was conducted on *D. angustifolia* using timberline-surviving as an informal control, with the following parameters (**Supplementary Table 34**): 6659 up-regulated DEGs and 7107 down-regulated DEGs (**Supplementary Figure 12A**, **Supplementary Table 35**). GO annotation was performed on the DEGs, and the secondary node of GO annotations for both up- and down-regulated DEGs included ‘response to stimulus’ (**Figure 5A**) in a similar manner. In response, the S/SR-strategy was adopted by the *D. angustifolia* in alpine tundra, which was different from the vast majority of native species that used the S or S/CS strategy (**Figure 2B**, **Supplementary Table 7**). This observation aligns with the prevailing understanding that the majority of invasive species employ R strategies to adapt to novel environments, while facing limitations imposed by the harsh tundra conditions (Cross et al., 2015; Rojas-Sandoval et al., 2022). The convergence of migrating species and most native species to S-selection related strategies in the harsh tundra environment, along with the similarity of CSR strategy types between neo-colonizers and most native species, suggests that both native and encroaching species may possess pre-adaptations to their respective local environments (Cornwell et al., 2006; Cross et al., 2015; Weiher and Keddy, 1999).

Notably, the ‘MAPK signaling pathway-plant (ko04016)’ was not annotated within the KEGG groups, but rather found in ‘plant-pathogen interaction (ko04626)’, ‘plant hormone signal transduction (ko04075)’, and ‘protein processing in endoplasmic reticulum (ko04141)’, among others (**Figure 5C**). Although LA did not exhibit a significant difference between the alpine tundra and timberline (**Supplementary Figure 12B**, **Supplementary Table 36**), PI-PLC displayed an upregulated gene (log2FC = 1.9). In this study, it was observed that proteins associated with self-regulated defense responses, including antioxidant enzymes (POD and CAT) and heat shock proteins (Hsp70, Hsp90, and Hsp20), exhibited an up-regulation in activity (**Figure 5B**, **Supplementary Table 37**). However, there was no evidence of cold stimulation, response to nitrogen starvation or water deficit, which aligns with the findings from the partial sequencing of native plants. The up-regulation of key enzyme genes was further confirmed through RT-qPCR analysis (**Figure 5D**).

## 4 Discussion

Our research investigates the morphological and molecular adaptative response of both indigenous species and encroaching species, providing partial validation of the gathered data. Furthermore, our study offers partial validation, challenges, and interpretations of prior findings. The results indicate that not all alpine tundra plants adhere strictly to the S strategy, but also exhibit the S/CS or S/SR strategy, or even the CS strategy, may be induced by a range of stimuli beyond temperature variations.

### 4.1 Morphological adaptations of native plants on tundra

Our research findings indicate that native species residing in alpine tundra display intraspecific trait variation (ITV) (Bjorkman et al., 2018), characterized by shorter plant heights and smaller leaf areas (**Supplementary Figures 1E, F**), when compared to species located at timberline. This observation suggests that native species in the alpine tundra have evolved to cope with the unique environmental conditions of this habitat and have developed a similar life form. This variation represents the environmental plasticity of plants rather than ecotypes (Xing et al., 2021), which ​has been widely verified​ in previous ecological research on Changbai Mountain (Chen et al., 2011a; Jin et al., 2018; Wang et al., 2019b; Zong et al., 2016b). Plants of similar life types exhibit shared characteristics and may utilize comparable adaptive strategies correlated with their environment. The challenging conditions of the alpine tundra environment played a role in preventing SLA from dominating in the differentiation of plant traits (**Figure 2A**). The occurrence of reduced plant height and leaf area in response to environmental stressors necessitates a reallocation of resources towards defense response, leading to a rebalancing of defense and growth strategies. The alpine tundra environment has led to the selection of phylogenetically distant species with similar life forms, resulting in phenotypic convergence that aids native plants in adapting to their surroundings. This convergence is also reflected in the adaptive strategies, particularly in defense responses, employed by plants.

### 4.2 Adaptive adjustment of native plants in molecular biology on alpine tundra

There is a possibility of considerable consistency in biological phenomena related to the acclimatization of indigenous species to alpine tundra. Examination of DEGs categorized under the level-two GO terms ‘catalytic activity’ and ‘response to stimulus’ suggests that plants may demonstrate a communal or distinct adaptive reaction to their surroundings. Furthermore, the scrutiny of level 3 GO terms for DEGs in 10 native species, particularly within the level-two GO term ‘response to stimulus’, unveiled 19 common terms (**Figure 3B**, **Supplementary Table 15**). This discovery implies that plants inhabiting the tundra biome may demonstrate unique adaptive reactions. Additionally, the KEGG annotation outcomes of DEGs suggest their participation in environmental adaptation pathways, such as the Mitogen-Activated Protein Kinase (MAPK) signaling cascade. The examination of DEGs has provided insights into the adaptive strategies utilized by plants in reaction to environmental stimuli. Native species of the alpine tundra exhibit defense response that involve the production of common proteins, such as Hsp70 and POD, to adapt to thermal stimuli and oxidative stress. This suggests that these species have developed highly specialized defense response, serving both as a driving force for adaptive adjustment to extreme environments and as a potential bottleneck in their response to climate change. Conversely, native species adapted to nitrogen deficiency, cold stimuli, and water deficit do not stimulate these common proteins but instead respond through respective patterns. These patterns are synthesized by plants as an optimal solution, balancing the differences in stimulus properties with resource allocation efficiency. These biological phenomena are evident in GO and KEGG annotations, as well as the convergence of biological processes.

The significant upregulation or downregulation of differential genes encoding identical proteins in tundra versus timberline environments represents a molecular adaptation strategy of plants to extreme alpine conditions, indicative of fundamental adjustments in energy allocation, defense response, and survival strategies (Zhang et al., 2024a). The downregulation of DEGs associated with proteins such as ​PRRs​, MYC2, ​ChiB, and ​PL-PLC reflects a critical ​growth-defense trade-off (Laxalt et al., 2025; Liu et al., 2025; Song et al., 2014; Wang et al., 2023; Zhai et al., 2013).​ Concurrently, the bidirectional expression changes in ​HSP70 and ​POD genes may be linked to energy reallocation under resource-limited stress (Huang et al., 2024; Wang et al., 2024).

### 4.3 Adaptation of encroaching species (*D. angustifolia*) on alpine tundra

In contrast to the native species, *D. angustifolia* exhibited no activation of MYC2 and ChiB at the molecular biology level. Additionally, only one up-regulated gene was observed in PI-PLC (log2FC = 1.9). However, it should be noted that this does not imply a lack of immune responses, thermal stimulus responsiveness, and antioxidation in *D. angustifolia* compared to native plants. Rather, *D. angustifolia* demonstrates a lower abundance of resistant protein types and fewer up-regulated DEGs. According to the ‘try harder’ theory, *D. angustifolia* is more aligned with the notion that invasive species are more prone to invading communities that lack similar species (Weiher and Keddy, 1999). This is attributed to the fact that invasive species exploit untapped resources and employ distinct resource acquisition strategies compared to native species (Ordonez et al., 2010; Rojas-Sandoval et al., 2022; Tecco et al., 2010). Consequently, this contributes to the further spread of *D. angustifolia* in alpine tundra ecosystems. At the transcriptome level, the annotation of DEGs reveals a limited number of proteins associated with self-regulated defense responses in comparison to native species. Notably, the ‘MAPK signaling pathway-plant (ko04016)’ remains unannotated. This lack of annotation suggests that *D. angustifolia* may not necessitate similar adaptive responses to cold, water scarcity, nitrogen starvation and other environmental stressors as observed in certain native species. This corresponds to the adoption of the S/SR strategy by *D. angustifolia* rather than the S or S/CS strategy adopted by native species, and the fact that the stresses imposed by the alpine tundra environment have not caused *D. angustifolia* to adopt a partly competitor but rather a ruderal strategy as other native species have done suggests that *D. angustifolia* has a greater adaptive capacity. Previous studies have only proved that the efficiency of nitrogen utilization of *D. angustifolia* is the direct cause of encroachment (Zong et al., 2016a).

**Figure 6:**
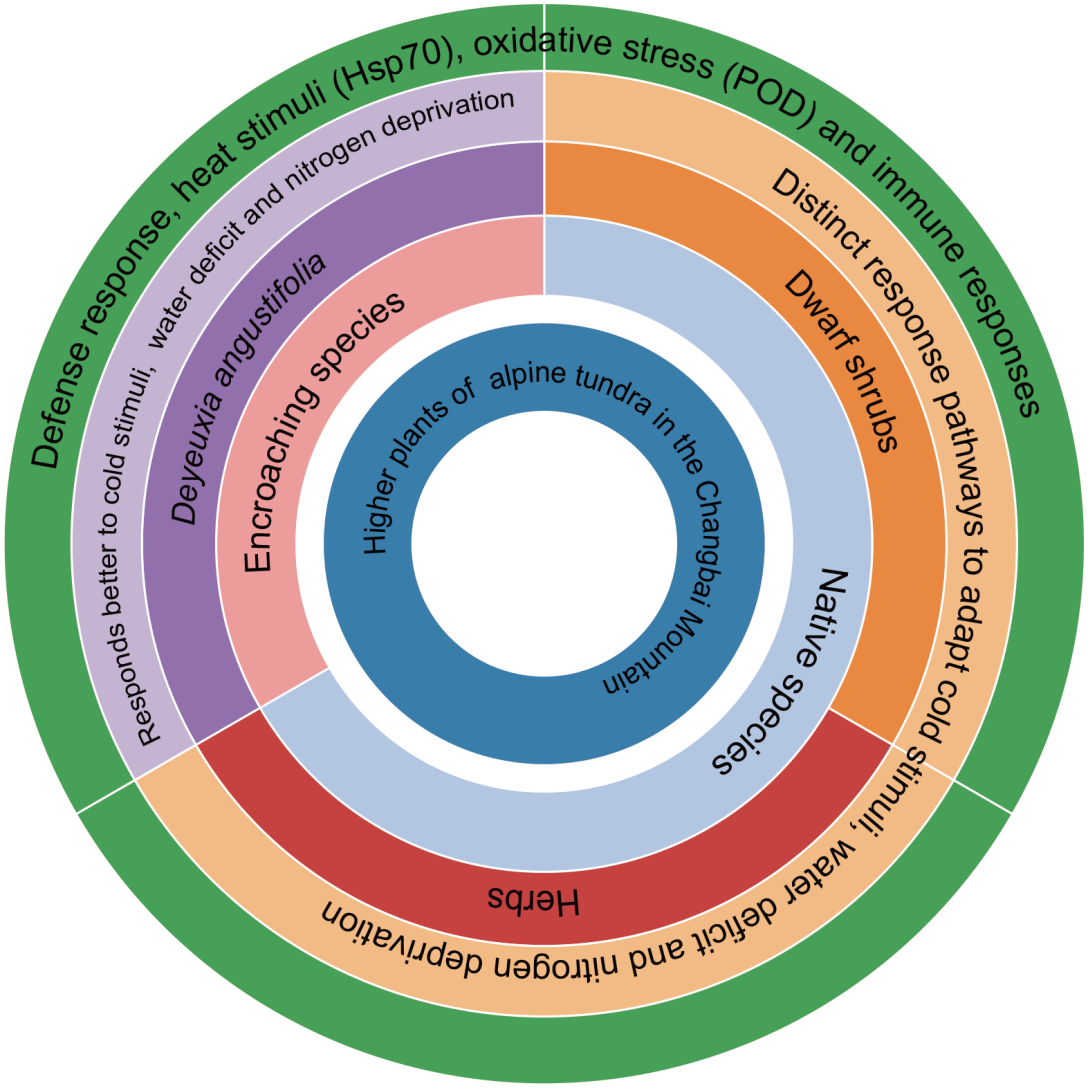
Summary diagram of higher plants adapted to the alpine tundra environment in Changbai Mountain. Parentheses represent co-activated proteins.

Our study does not deny this view, but also considers that the high efficiency of nitrogen starvation and adaptability to water shortage and insensitivity to cold stimulation are the main reasons for the spread of *D. angustifolia* in alpine tundra (**Figure 6**). This finding aligns with the notion that successful invasive species often exhibit superior adaptation to local environments compared to native species (Lowry et al., 2013). The findings of various studies indicate that the presence of epigenetic variation and DNA methylation plays a significant role in facilitating the adaptive adjustment of *D. angustifolia* to alpine tundra environments (Ni et al., 2021). Moreover, our own study provides additional evidence suggesting that *D. angustifolia* exhibits superior molecular-level adaptation compared to native species.

### 4.4 Adaptation trends of plants in alpine tundra

The alpine tundra, serving as the uppermost elevation threshold for the growth of higher plants, represents an extremely challenging habitat for plant adaptive response. Nevertheless, the CSR strategy analysis has revealed that certain herbaceous species and *R. aureum* do not strictly adhere to the S strategy, but rather exhibit strategies related to S. This suggests that there remains potential for these species to acclimate to more severe tundra conditions, which is also consistent with the transition from alpine shrub tundra to herbaceous tundra in Changbai Mountain (Jin et al., 2019; Wang et al., 2019b).

Analyses of native species indicated that these species have adapted to nitrogen, water, and cold-deficient conditions without activating the associated common proteins. This suggests that such stimuli are less restrictive for plants in alpine tundra environments compared to adaptation to oxidative stress and thermal stimuli. Similarly, encroaching species (*D. angustifolia*) did not exhibit adaptive sensitivity to nitrogen, water, and cold-deficient conditions while adapting to other stimuli. This observation may be associated with the ongoing ecological niche reconfiguration in the alpine tundra of Changbai Mountain driven by climate change. The transcriptional regulatory pressure exerted by traditional stressors (e.g., nitrogen deficiency, water deficit, and cold conditions) on plants may be lower than that of other stressors, and the association between gene expression patterns and species expansion phenomena suggests that traits related to resource acquisition may be one of the potential factors influencing the ecological niche reconfiguration of this species in the tundra (**Figure 6**). Herbaceous plants represented by *D. angustifolia* and *Veratrum oxysepalum* have encroached upward into the alpine tundra of Changbai Mountain (Lei et al., 2025; Zong et al., 2016a), and the further expansion of herb-dominated tundra (Wang et al., 2019b). This phenomenon is consistent with the broader pattern of global warming, which has led to more complex and variable precipitation patterns during the alpine flora growing season, thereby influencing the water and nitrogen availability and the native species’ adaptive adjustment to water stress (Maes et al., 2024; Malfasi and Cannone, 2020).

### 4.5 Convergence of alpine tundra plant adaptations in Changbai Mountain

Our study involved the synthesis of CSR strategy and transcriptomic studies of dwarf shrubs, herbs, and encroaching species. We discovered that all plant species examined implemented S-related strategies, with native species exhibiting defense response, oxidative stress responses, immune system activation, reactions to thermal stimuli, adaptive response to nitrogen deprivation, and varying levels of tolerance to water scarcity. Similarly, encroaching species also displayed defense response, responses to thermal stimuli, and oxidative stress reactions, indicating a convergence of adaptive strategies in alpine tundra vegetation. The validation of the CSR strategy was confirmed, as evidenced by the co-activation of proteins at the molecular level by both native plants and the encroaching species. While prior research has predominantly emphasized phenotypic characteristics (Bjorkman et al., 2018; Mumbanza et al., 2022), our study makes a significant contribution to the progression of knowledge in the study of challenging environments. Our research enhances current knowledge of how native plants in alpine tundra respond to environmental changes and investigates the factors contributing to *D. angustifolia*’s adaptation in the context of global climate change. These results offer valuable insights for future studies on alpine plant ecology in the face of understanding the adaptations and ecological relationships of species in alpine tundra ecosystems. The confounding effects of multiple environmental variables cannot be completely ruled out in this research, and subsequent experiments are required to verify the causal relationship. This study only compared the transcriptomic differences between the current alpine tundra and timberline habitats, and thus cannot reflect the temporal dynamic changes of stress factors (e.g., nitrogen availability, low-temperature intensity). It can only infer the current priority of stress responses based on gene expression patterns. Additionally, this study provides a transcriptome-level hypothesis for the adaptation and upward encroachment response of alpine tundra species, and the causal relationships involved await verification by subsequent controlled experiments.

## 5 Conclusion

The adaptive adjustment of native plants in the alpine tundra of Changbai Mountain exhibit convergent. These plants must develop defense response against thermal stimuli, oxidative stress, and immune responses, as well as adapt to nitrogen starvation and varying levels of water scarcity. However, the extent to which species need to adapt to cold stimuli may differ among plant species from the transcriptional level. These findings elucidate the adoption of S-related strategies by native tundra plants. The efficient adaptation to nitrogen deficiency, tolerance to water deficits, and insensitivity to cold stimuli are likely key factors contributing to the proliferation of *D. angustifolia* in alpine tundra environments. The transcriptional regulatory pressure exerted by traditional stressors (e.g., nitrogen deficiency, water deficit, and cold conditions) on plants may be lower than that of other stressors, and the association between gene expression patterns and species expansion phenomena suggests that traits related to resource acquisition may be one of the potential factors influencing the ecological niche reconfiguration of this species in the tundra.

## Data Availability

The datasets presented in this study can be found in online repositories. The names of the repository/repositories and accession number(s) can be found in the article/**Supplementary Material**.
