# Supplementary material for: Divergent functional traits and gene expression profiles in native and encroaching plant species across an alpine elevational gradient
Source: Front Plant Sci. 2025 Oct 7;16:1656812. doi: 10.3389/fpls.2025.1656812 (PMC12539351; doi:10.3389/fpls.2025.1656812)
Supplement: Supplementary file 1 [file DataSheet1.docx]

**SUPPLEMENTARY FIGURES**

**Dynamics of greenhouse gases in forest systems**

Zhongzan Yang^1^, Jian You^1^, Jiangnan Li^1^, Wei Zhao^1^, Ming Xing^1^, Jiaxin Zhao^1^, Yujiao Zhang^1^, Ma Cui^1^, Yuqiao Gong^1^, Yueming Zhao^1^, Xia Chen^1^

1. National & Local United Engineering Laboratory for Chinese Herbal Medicine Breeding and Cultivation, School of Life Sciences, Jilin University, Changchun, Jilin Province, China

Corresponding author:

Xia Chen^1^

E-mail: chenxiajlu@163.com


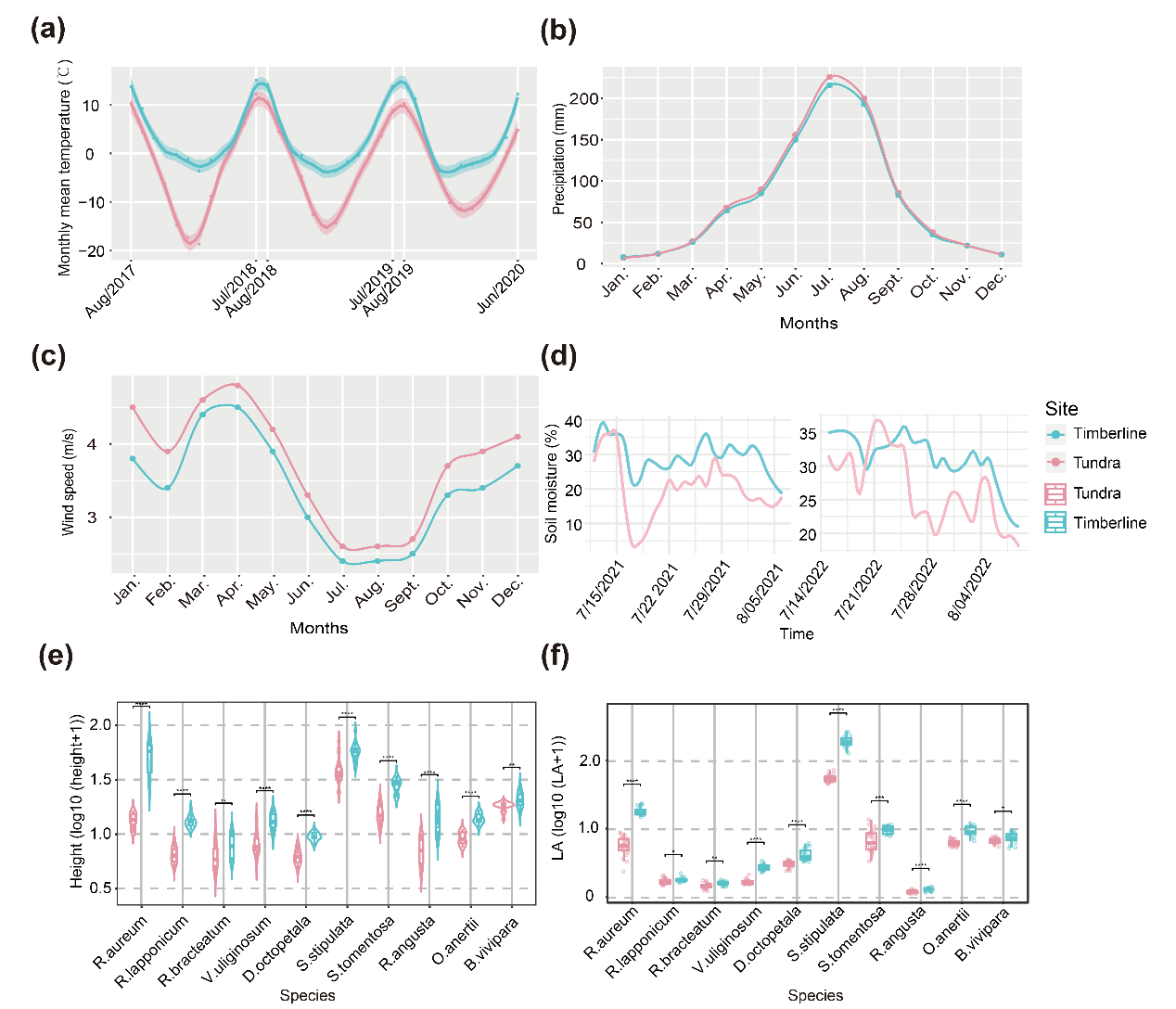


**Supplementary Figure 1** Climate data and plant phenotypes. Climate data were downloaded from WorldClim ([www.worldclim.org](http://www.worldclim.org)). (**a**) Monthly mean temperature (10cm below ground) for 2017-2020 in tundra and timberline. Color bands represent the 95% confidence. (**b**) Average monthly precipitation for alpine tundra and timberline from 1970 to 2000. (**c**) Average monthly wind speed for alpine tundra and timberline from 1970 to 2000. (**d**) Soil moisture content of tundra and timberline from mid-July to early August, 2021-2022. (**e**) Plant height of 10 native plants. The T-test was used to evaluate the significance of differences (*** *P* < 0.001; ** *P* < 0.01; * *P* < 0.05, the below is the same). In violin plots, the horizontal bars represent medians. The tops and bottoms of the boxes show the 75 th and 25 th percentiles, respectively (the below is the same). (**f**) Leaf area (LA) of 10 native plant species.


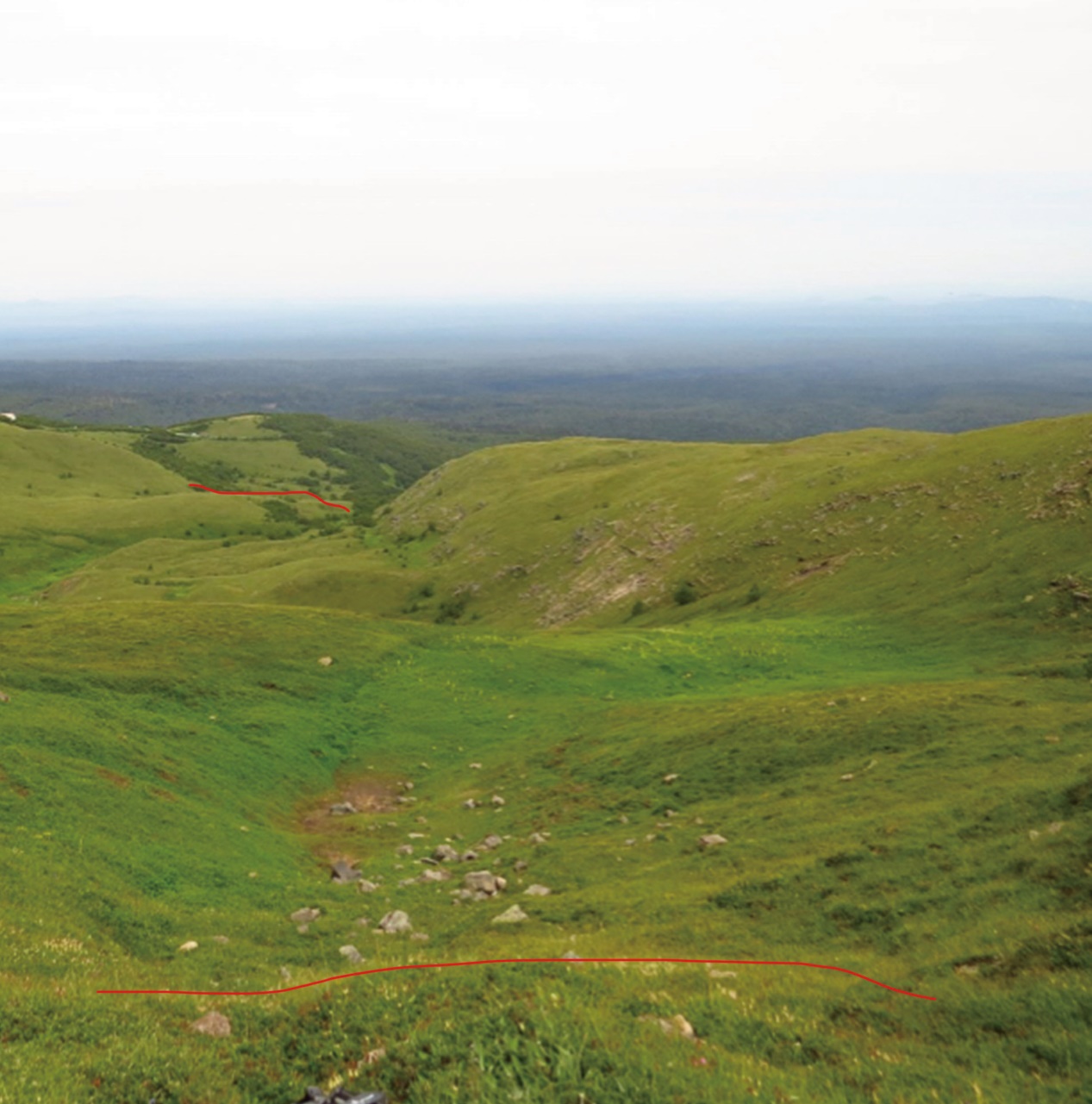


**Supplementary Figure 2** A portion of the tundra and timberline sampling sites. The red lines show the approximate locations of the sample.


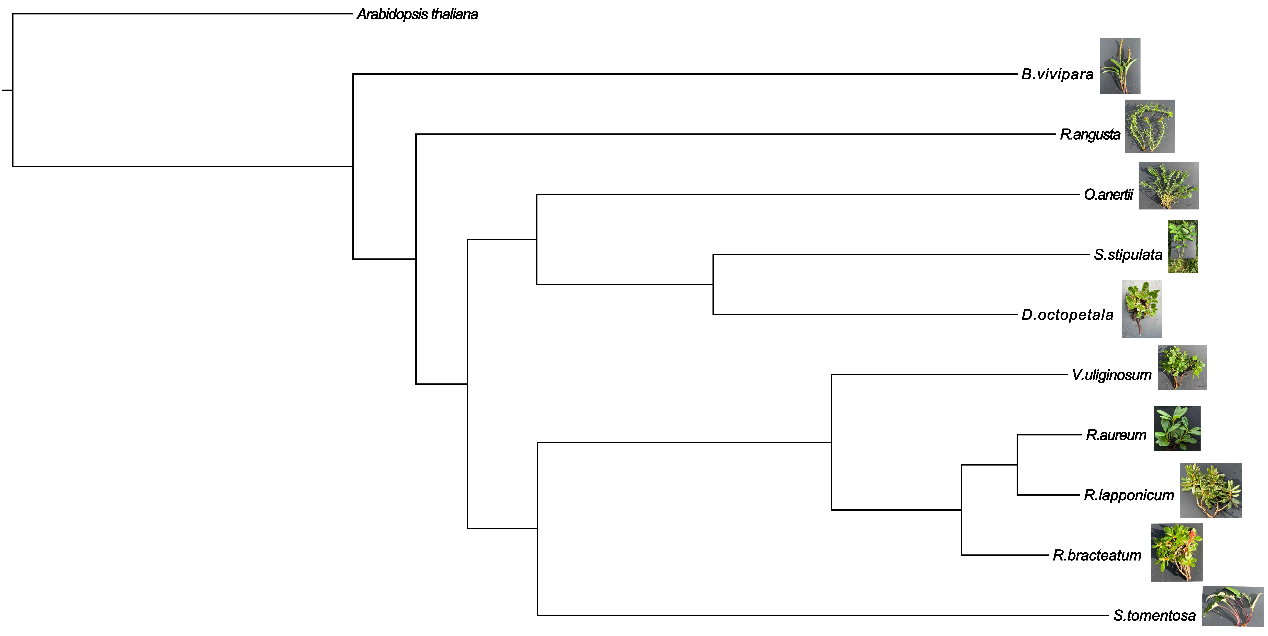


**Supplementary Figure 3** Phylogenetic trees for 10 native species in Changbai mountain alpine tundra. Pictures show specific species morphology.

**
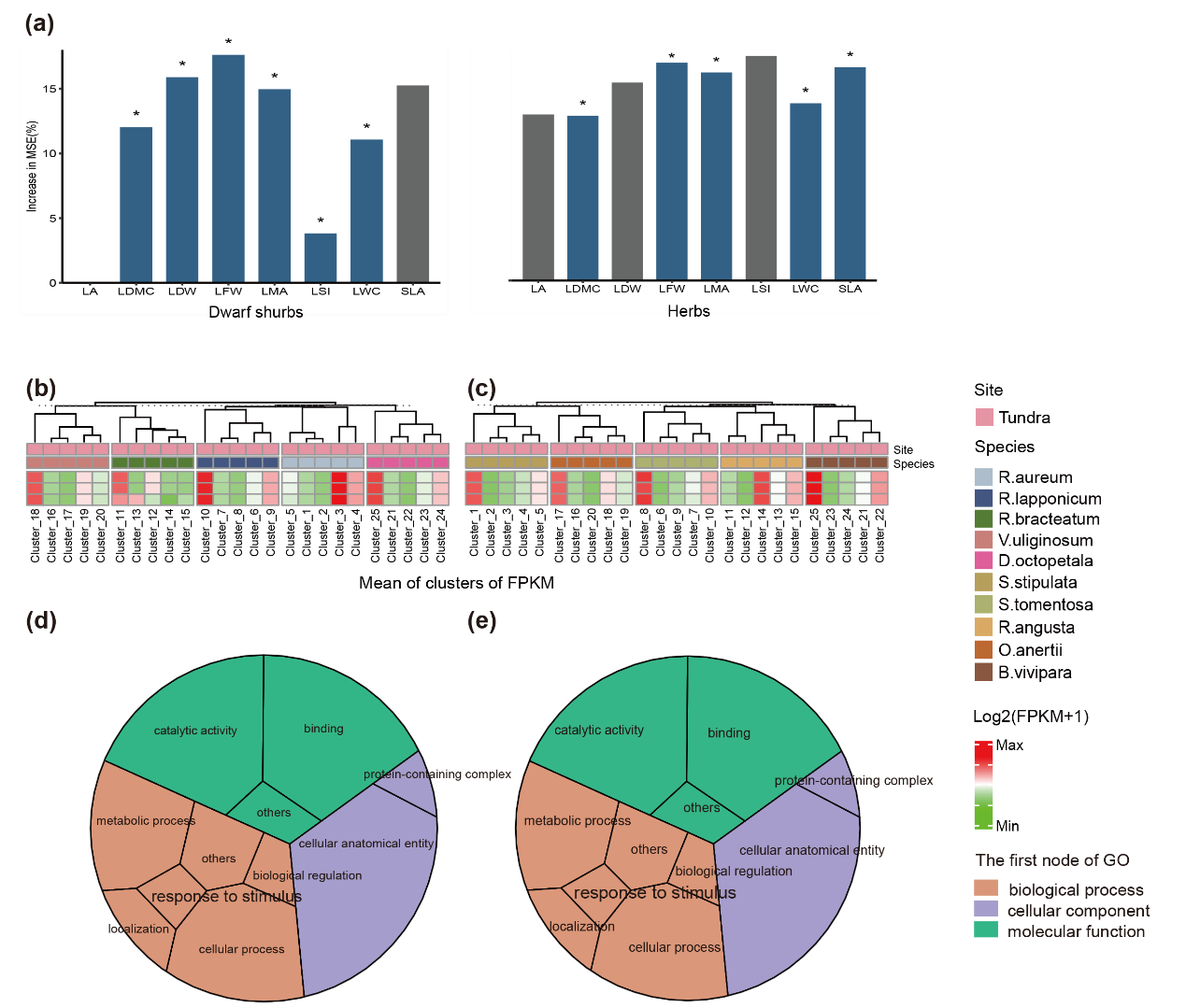
**

**Supplementary Figure 4** (**a**) Random forest (RF) predicts the importance of leaf traits of dwarf shrubs and herbaceous plants on S scores of CSR strategy. Genes expression of dwarf shrubs (**b**) and herbs (**c**) in alpine tundra grouped by Pearson correlation hierarchical clustering on the mean valued of each cluster clustered by K-medoids. A total of 40,000 annotated genes per species were subjected to random standardization and subsequently clustered using K-medoids algorithm, with the maximum number of clusters set to K=5 based on habitat information derived from logarithmic FPKM values. Pearson correlation hierarchical clustering was then conducted on the mean values of each cluster, divided into three groups. GO terms of genes in groups of highest FPKM values (first clustered into 5 clusters by K-medoids and then Pearson correlation hierarchical clustering) for dwarf shrubs (**d**) and herbs (**e**) on tundra. There was no statistically significant distinction observed in the gene annotation statistics pertaining to the indexing of the secondary node of GO for dwarf shrubs and herbaceous species in alpine tundra (Pearson's chi-squared test, *p* > 0.05).


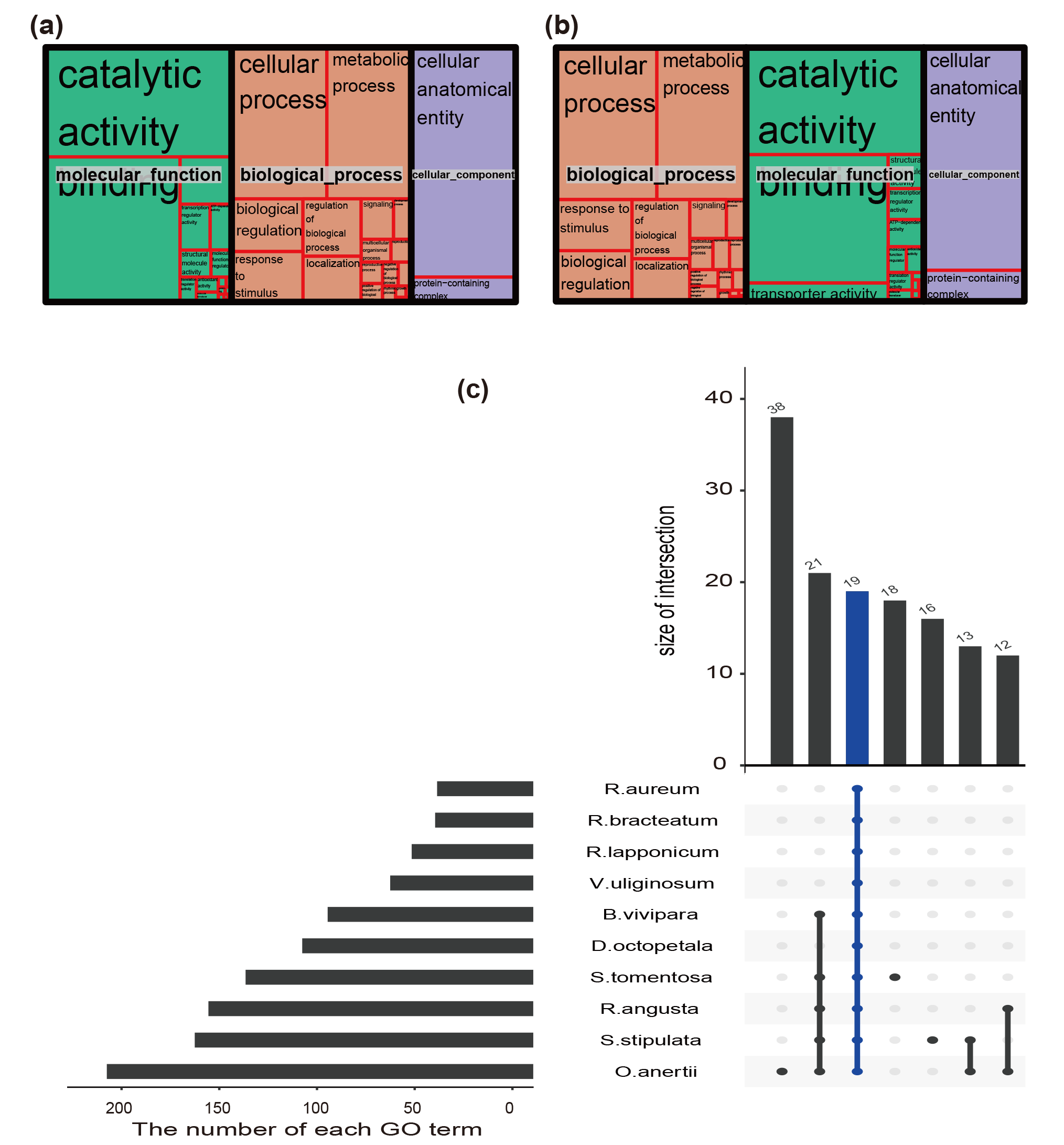


**Supplementary Figure 5** GO terms of DEGs in dwarf shrubs (**a**) and herbs (**b**). There were no statistically significant differences in the percentage of level 2 GO term annotations between dwarf shrubs and herbs within the two groups (Pearson's chi-squared test, *p*＞0.05). (**c**) Partial UpSet analysis of the number of GO terms and their intersection sizes for 10 native plant species. The horizontal bars show the total number of GO terms for each species and the vertical bars illustrate the number of GO terms in common (intersection size). The blue color represents the intersection of ten species annotations.


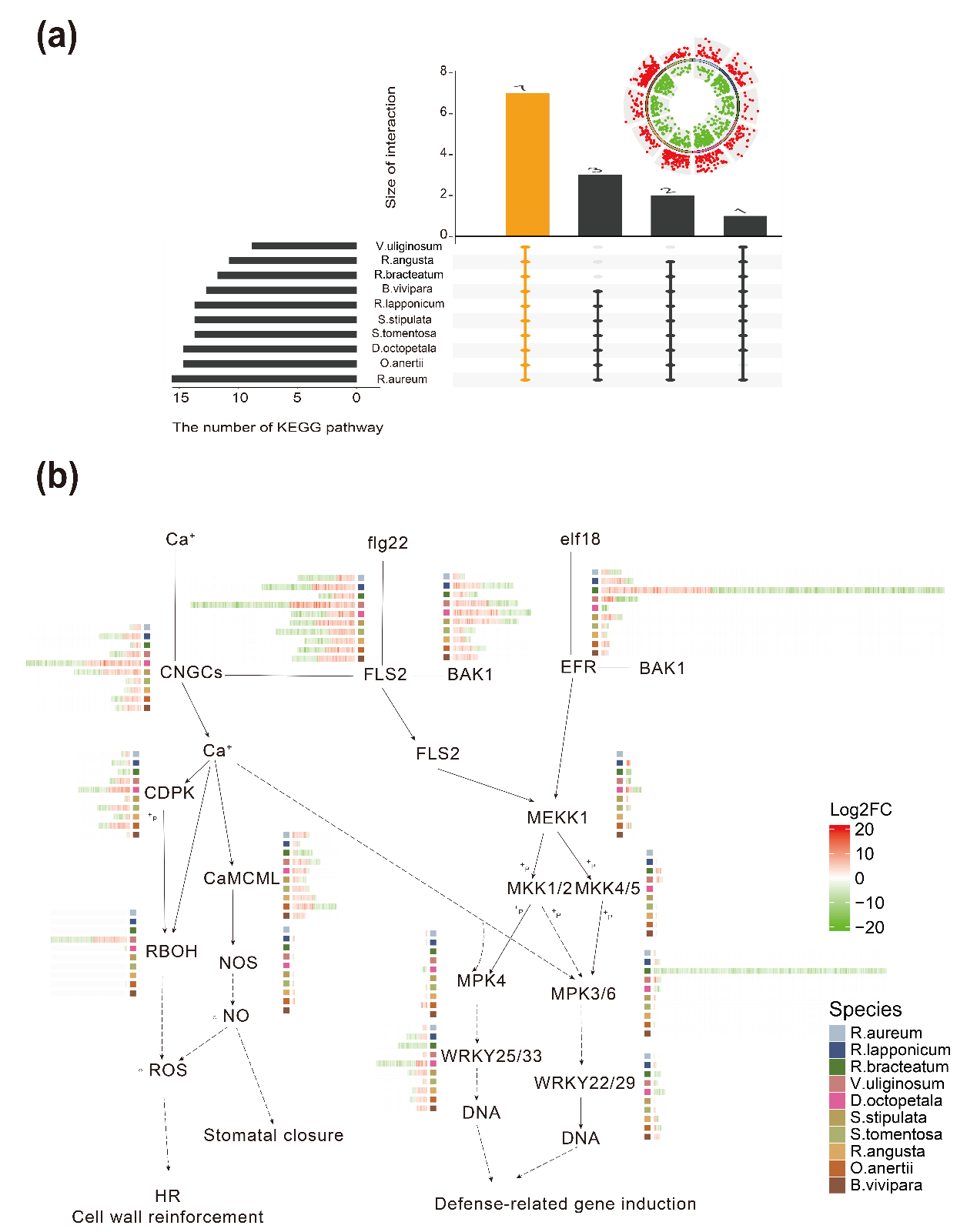


**Supplementary Figure 6** (**a**) Circle volcano plot shows ten species’ DEGs which are significantly upregulated (red dots) or downregulated (green dots) annotated by GO term ‘defense response (GO:0006952)’ and partial UpSet analysis of the number of KEGG annotations of DEGs annotated to GO terms ‘defense response (GO:0006952)’ and their intersection sizes for 10 native plant species. The horizontal bars show the total number of KEGG annotations for each species and the vertical bars illustrate the number of KEGG annotations in common (intersection size), the same below. The orange color represents the intersection of ten species annotations. (**b**) Partial PAMP or MAMP-triggered immunity for 10 native species in alpine tundra.

**
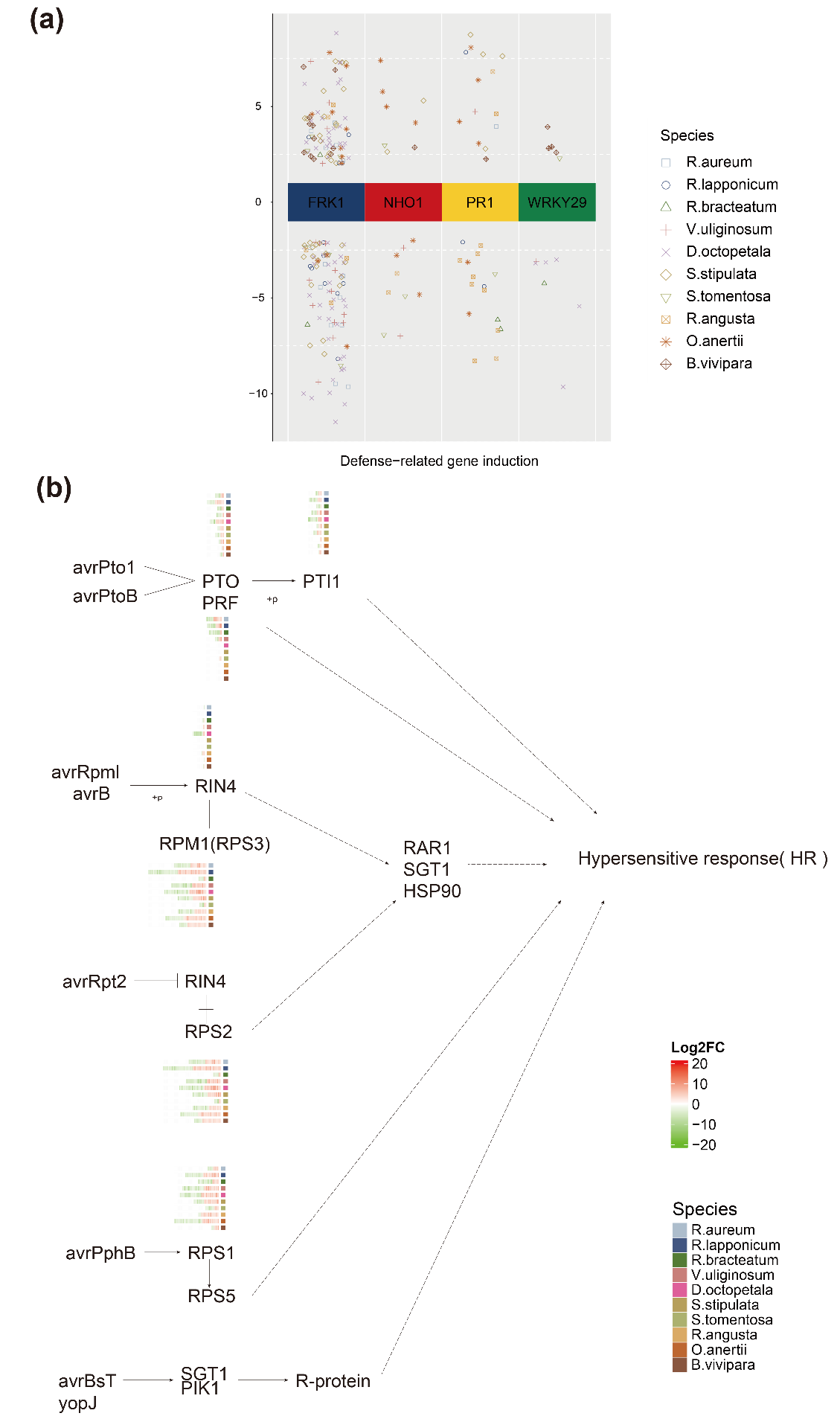
**

**Supplementary Figure** **7** (**a**) Partial defense-related DEGs induction. Different shapes and color combinations represent different species. (**b**) Partial Effector-triggered immunity for 10 native species in alpine tundra.


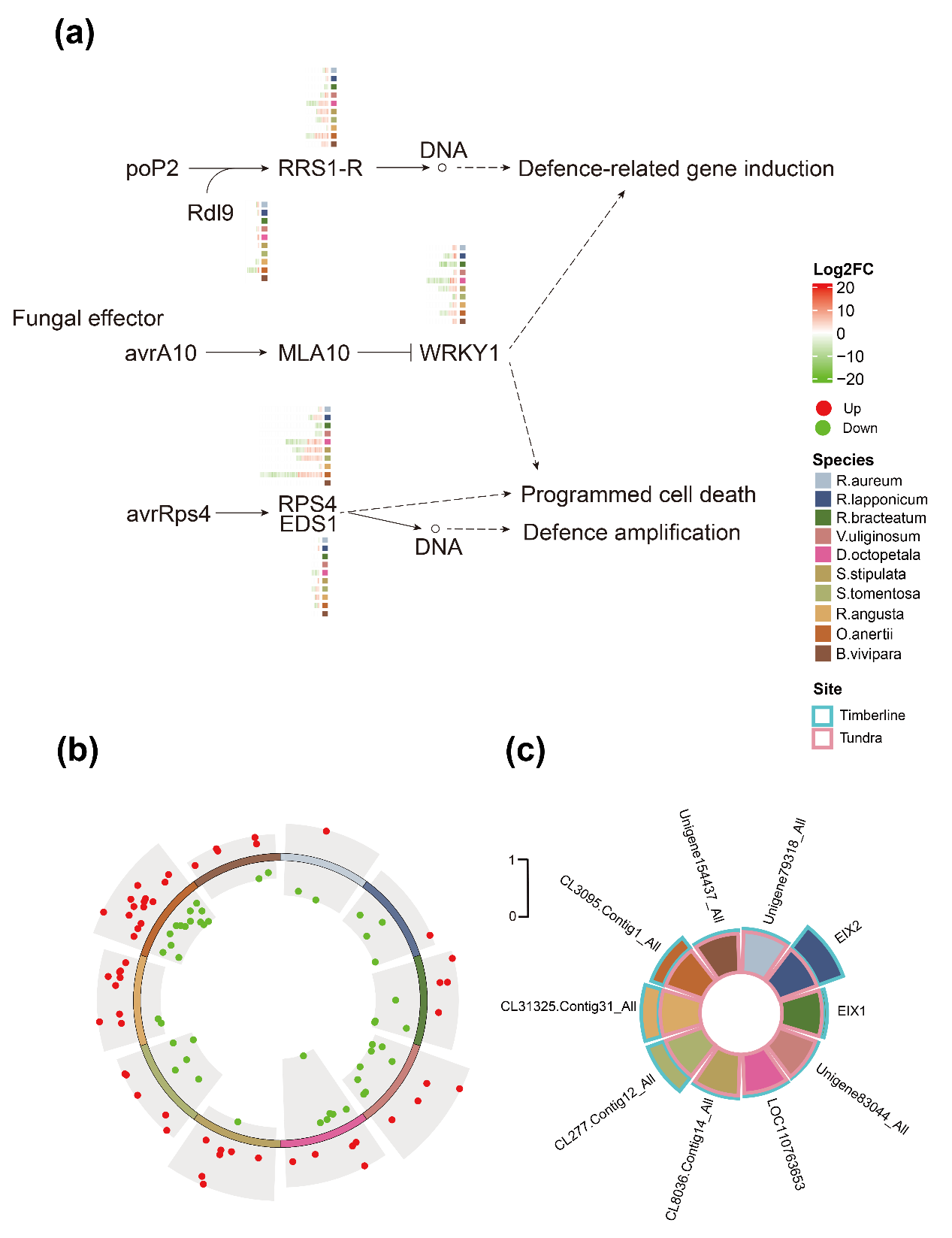


**Supplementary Figure 8** (**a**) Partial Effector-triggered immunity for 10 native species in tundra. (**b**) Ten species annotated to DEGs for KCS. Circle volcano plots showed log2FC for DEGs which are significantly upregulated (red dots) or downregulated (green dots), the same below. (**c**) Relative expression of the genes related to EIX1/2 by RT-qPCR.


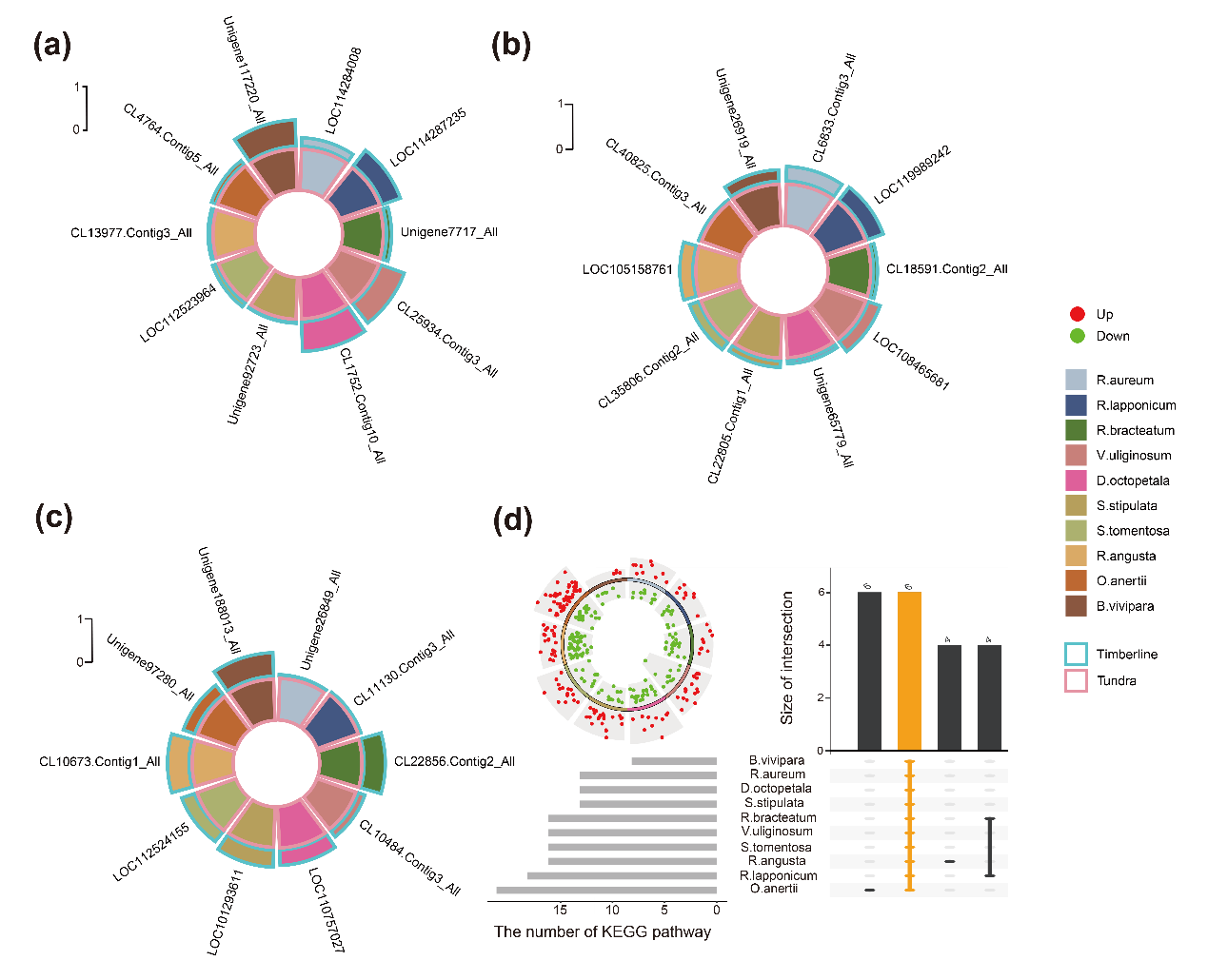


**Supplementary Figure 9** Relative expression of the genes related to MYC2 (**a**), ChiB (**b**), PI-PLC (**c**) by RT-qPCR. (**d**) Circle volcano plot shows ten species’ DEGs annotated by GO term ‘response to oxidative stress (GO:0006979)’ and partial UpSet analysis of the number of KEGG annotations of DEGs annotated to GO terms ‘response to oxidative stress (GO:0006979)’ and their intersection sizes for 10 native plant species. The orange color represents the intersection of ten species annotations.


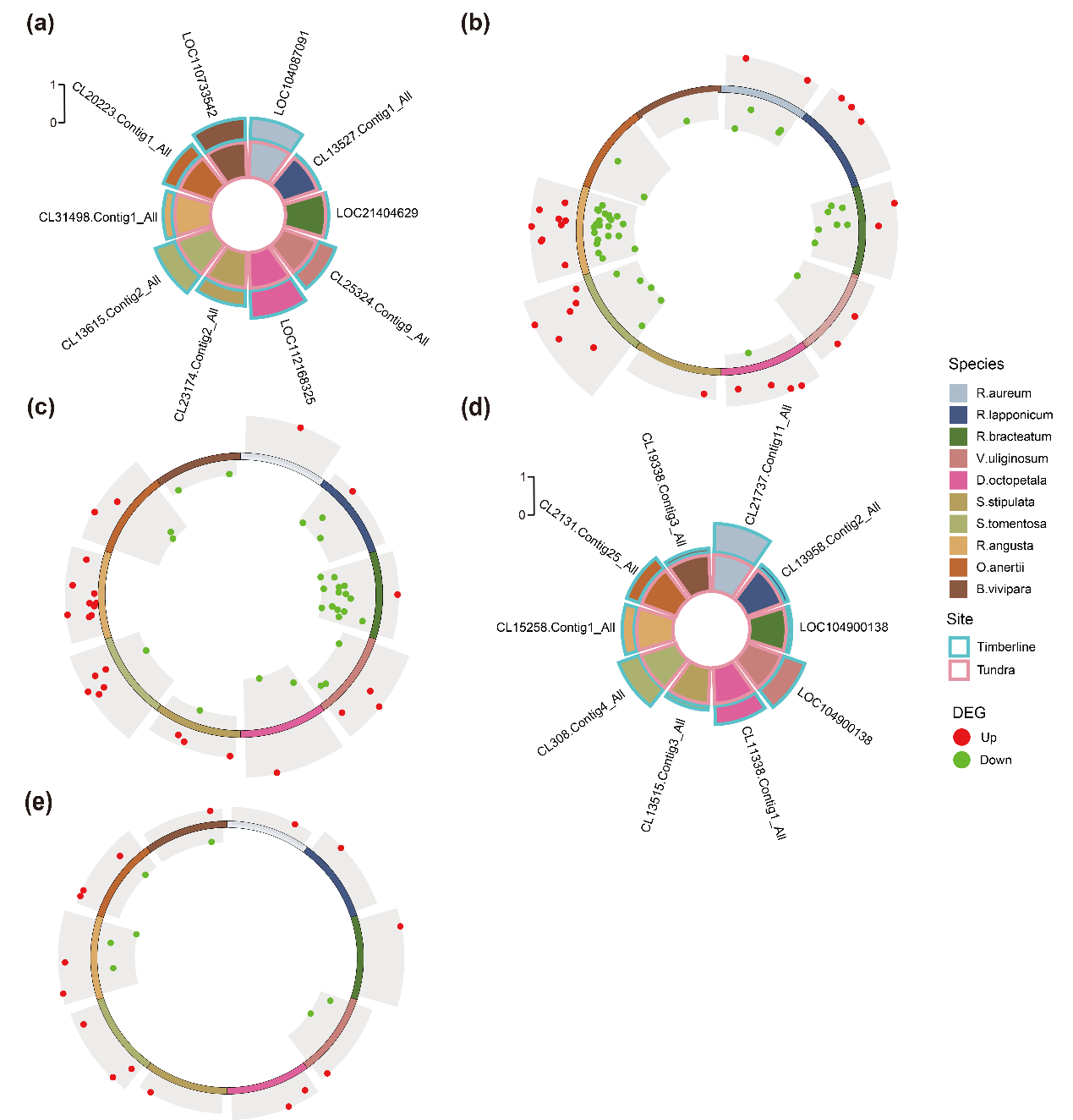


**Supplementary Figure 10** Relative expression of the genes related to POD (**a**) and Hsp70 (**d**) by RT-qPCR. Ten species annotated to DEGs for CAT (**b**) and GPX (**c**). (**e**) DEGs annotated to GO term ‘cellular response to nitrogen starvation (GO:0006995)’.


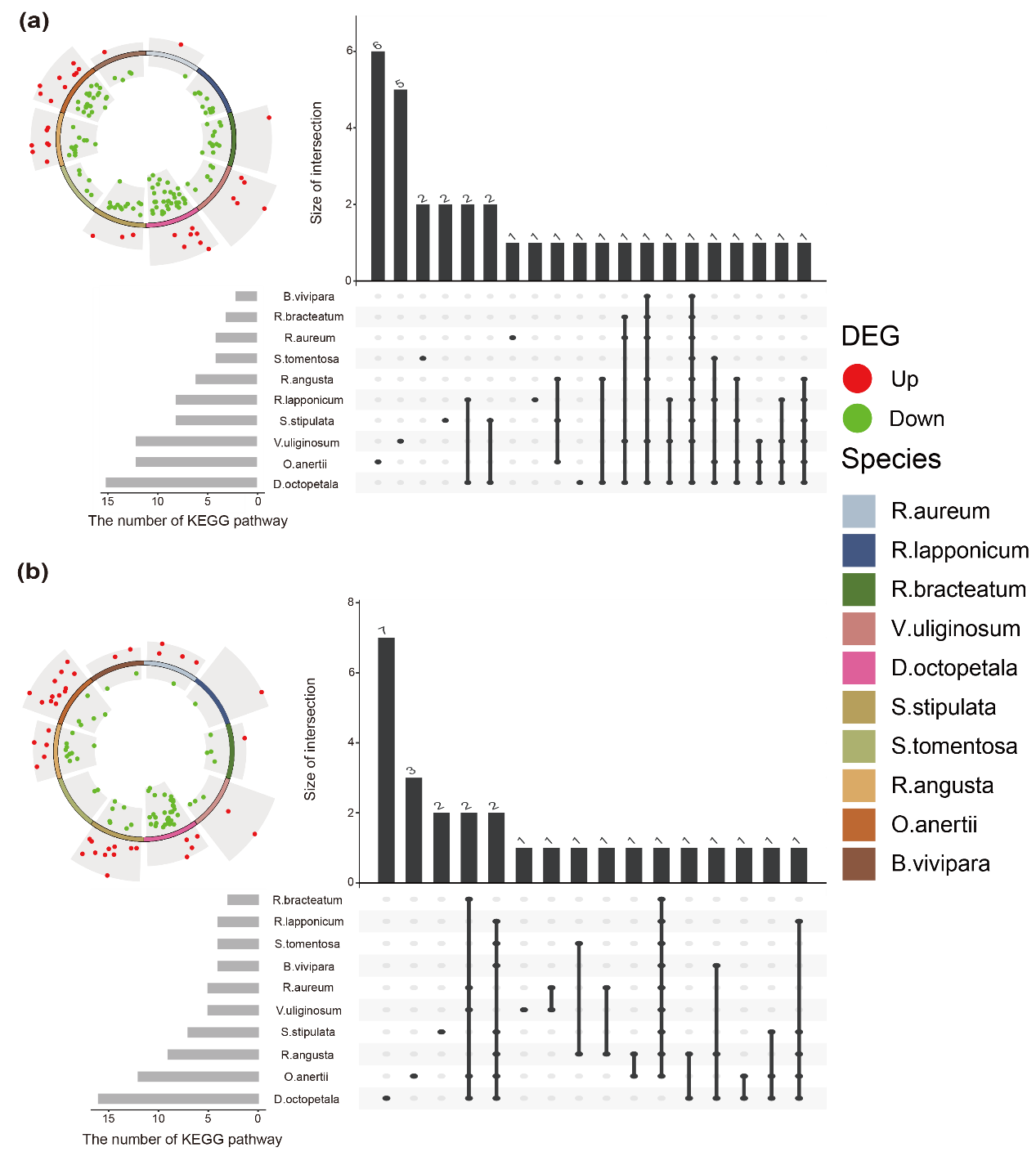


**Supplementary Figure 11** (**a**) Circle volcano plot shows ten species’ DEGs annotated by ‘response to cold (GO:0009409)’ and partial UpSet analysis of the number of KEGG annotations of DEGs annotated to GO terms ‘response to cold (GO:0009409)’ and their intersection sizes for 10 native plant species. (**b**) Circle volcano plot shows ten species’ DEGs annotated by ‘response to water deprivation (GO:0009414)’ and partial UpSet analysis of the number of KEGG annotations of DEGs annotated to GO terms ‘response to water deprivation (GO:0009414)’ and their intersection sizes for 10 native plant species.

**
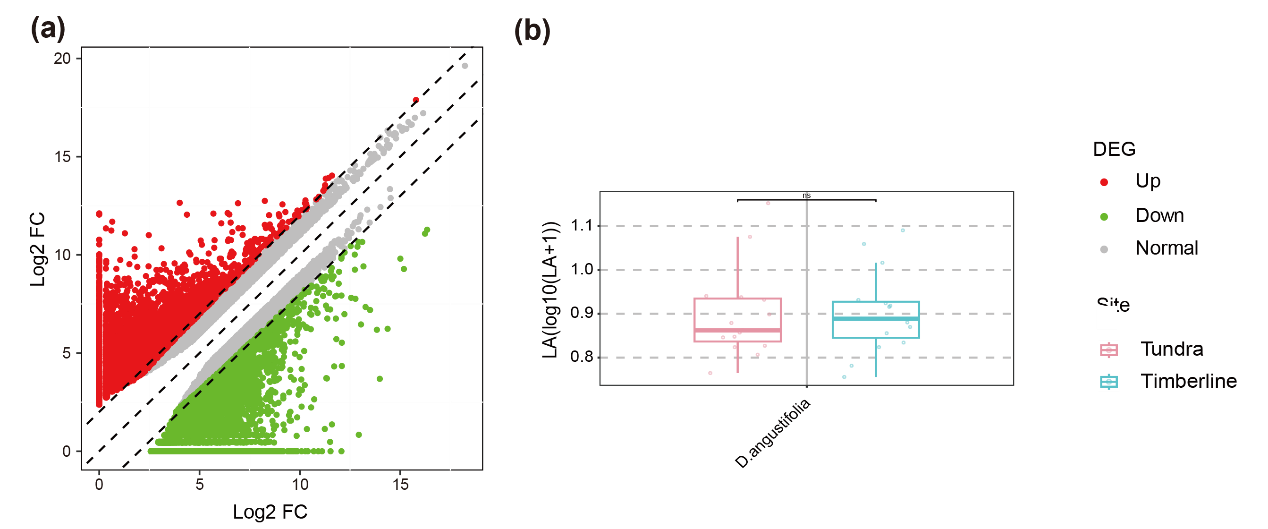
 Supplementary Figure 12** (**a**) Distribution of DEGs in *D.angustifolia*. (**b**) Leaf area (LA) of *D.angustifolia*.
